# Supplementary material for: Accurate and complete genomes from metagenomes
Source: Genome Res. 2020 Mar;30(3):315–33. doi: 10.1101/gr.258640.119 (PMC7111523; doi:10.1101/gr.258640.119)
Supplement: Supplemental Material [file supp_30_3_315__index.html]

Accurate and complete genomes from metagenomes — Supplemental Material 

# Accurate and complete genomes from metagenomes

## Supplemental Material

- Supplemental\_Fig\_S1.pdf
- Supplemental\_Fig\_S2.pdf
- Supplemental\_Fig\_S3.pdf
- Supplemental\_Fig\_S4.pdf
- Supplemental\_Fig\_S5.pdf
- Supplemental\_Fig\_S6.pdf
- Supplemental\_Fig\_S7.pdf
- Supplemental\_Fig\_S8.pdf
- Supplemental\_Fig\_S9.pdf
- Supplemental\_Fig\_S10.pdf
- Supplemental\_Fig\_S11.pdf
- Supplemental\_Methods.pdf
- Supplemental\_Table\_S1.xlsx
- Supplemental\_Table\_S2.xlsx
- Supplemental\_Table\_S3.xlsx
- Supplemental\_Table\_S4.xlsx
- Supplemental\_Table\_S5.xlsx
- Supplemental\_Table\_S6.xlsx
- Supplemental\_Table\_S7.xlsx
- Supplemental\_Table\_S8.xlsx
