# Supplementary material for: Accurate and complete genomes from metagenomes
Source: Genome Res. 2020 Mar;30(3):315–33. doi: 10.1101/gr.258640.119 (PMC7111523; doi:10.1101/gr.258640.119)
Supplement: Supplemental Material [file supp_gr.258640.119_Supplemental_Fig_S1.pdf]

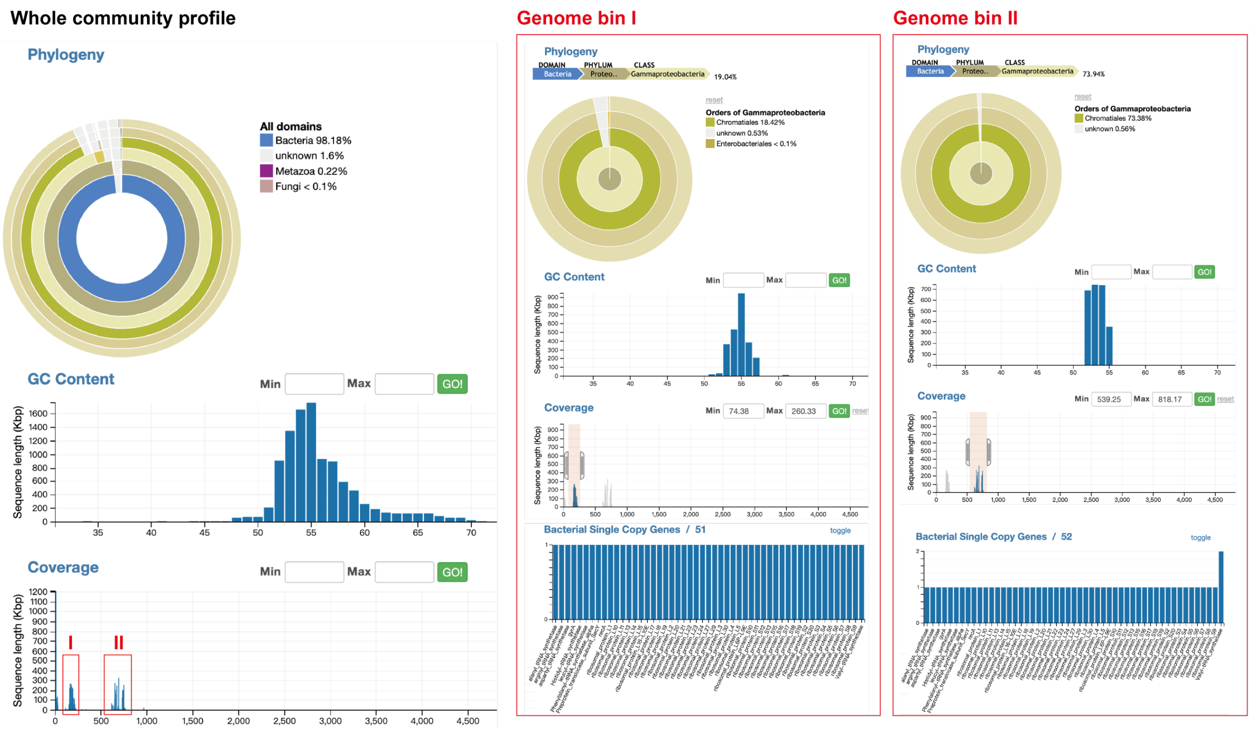

Supplemental Fig S1. Example of bin establishment from a simple microbial community. The two most abundant genomes share similar GC content but the coverage profiles differ so the scaffolds can be easily separated based on coverage. The phylogenetic profile, GC content of the two sets of contigs with the selected coverage ranges and inventory of expected single-copy genes are shown on the right.
