# Supplementary material for: Accurate and complete genomes from metagenomes
Source: Genome Res. 2020 Mar;30(3):315–33. doi: 10.1101/gr.258640.119 (PMC7111523; doi:10.1101/gr.258640.119)
Supplement: Supplemental Material [file supp_gr.258640.119_Supplemental_Fig_S2.pdf]

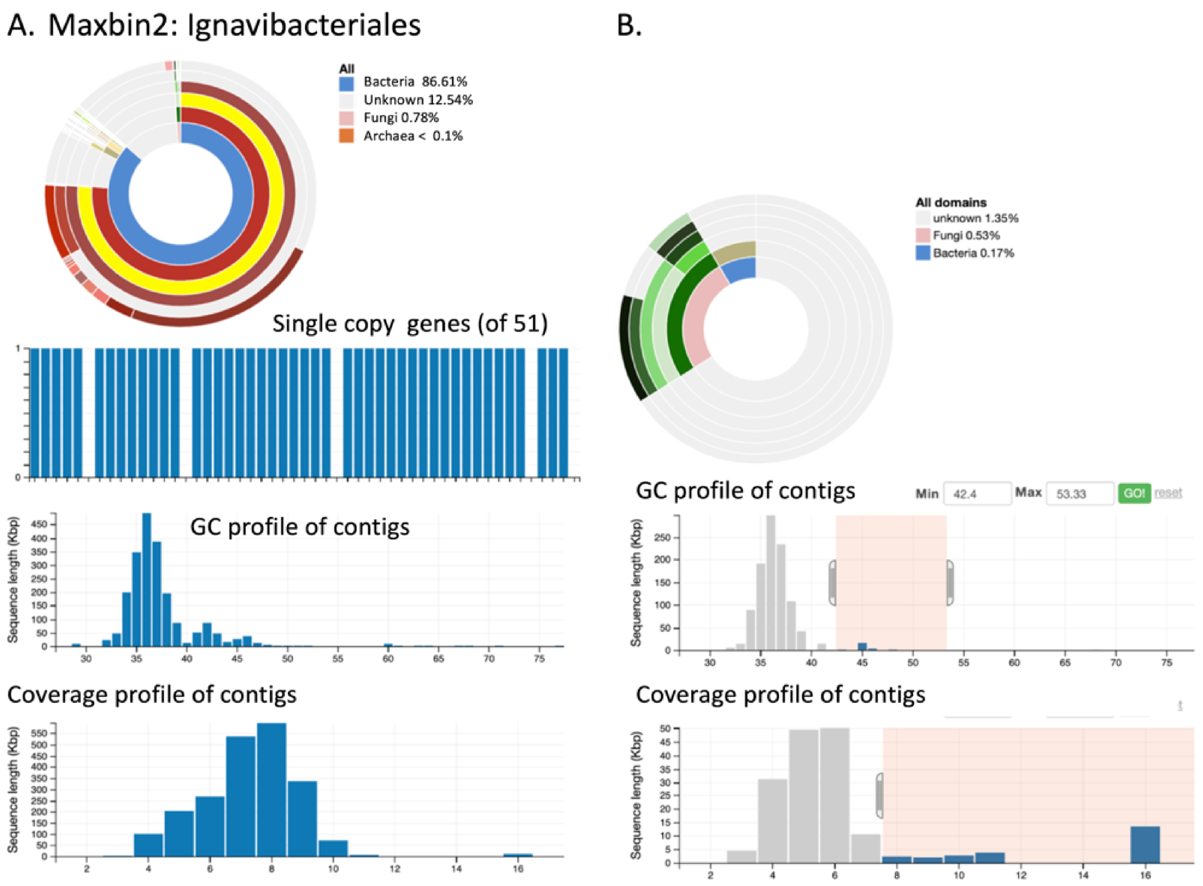

Supplemental Fig S2. Overview of a draft genome generated by autobinning. The DASTool algorithm selected a bin made using Maxbin2. However, like many automatically generated bins, the draft genome contains contaminating sequences that are not evident based on the single-copy gene inventory. **A.** Phylogenetic profile (wheel profiling overall contig set at various taxonomic levels, from Domain (inner ring) to species (outermost ring)). Note the dominance of the bin by sequences profiled as Ignavibacteriales but the presence in the bin of sequences from other phyla and domains and without a domain profile (Unknown), peaks with anomalously high GC and some sequence with higher than normal coverage. A 56 kbp phage sequence was removed from the Unknown domain **B.** One step in bin curation: the selection of a subset of scaffolds with anomalous coverage and GC content indicates the presence in the bin of eukaryotic and other contaminants.
