# Supplementary material for: Accurate and complete genomes from metagenomes
Source: Genome Res. 2020 Mar;30(3):315–33. doi: 10.1101/gr.258640.119 (PMC7111523; doi:10.1101/gr.258640.119)
Supplement: Supplemental Material [file supp_gr.258640.119_Supplemental_Fig_S3.pdf]

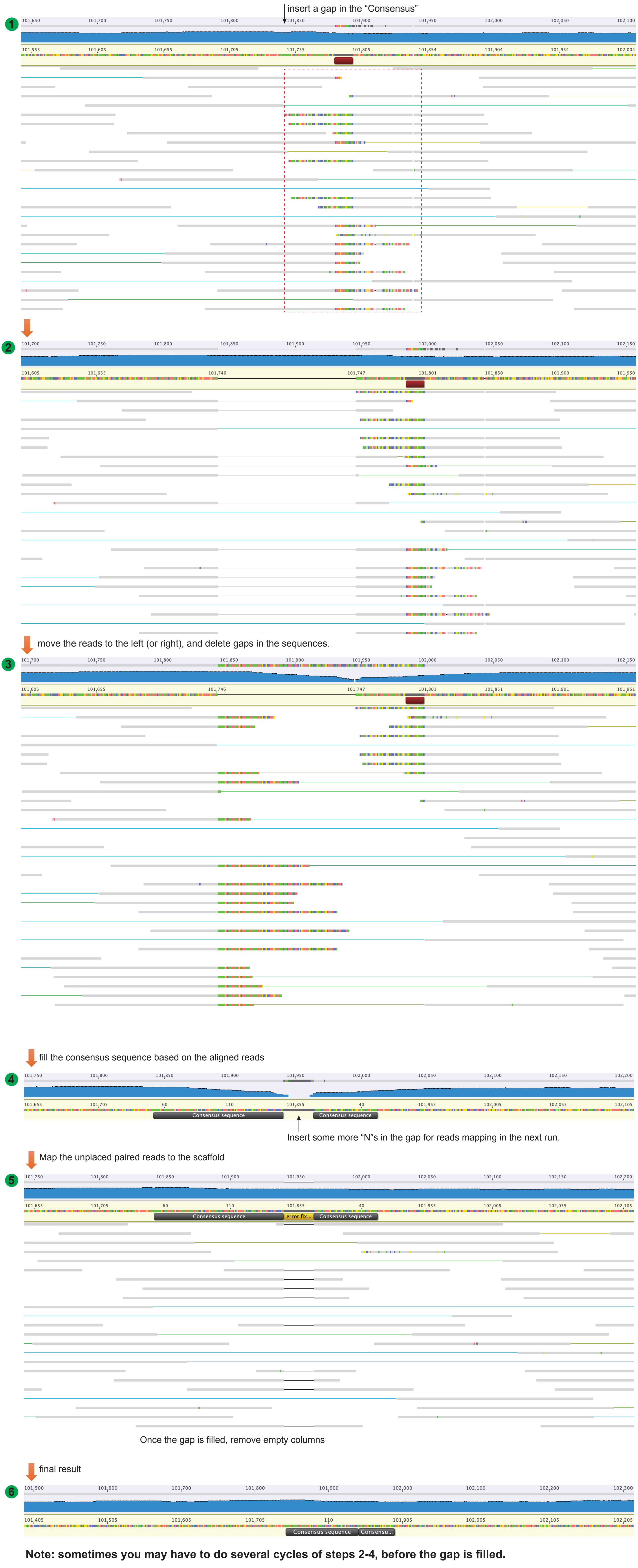

Supplemental Fig S3. Diagrams showing the procedure used to fix a local assembly error using unplaced paired-end reads mapped to the problem region after opening a gap.
