# Supplementary material for: Accurate and complete genomes from metagenomes
Source: Genome Res. 2020 Mar;30(3):315–33. doi: 10.1101/gr.258640.119 (PMC7111523; doi:10.1101/gr.258640.119)
Supplement: Supplemental Material [file supp_gr.258640.119_Supplemental_Fig_S4.pdf]

Ns were wrongly inserted during scaffolding. The sequences flanking the Ns are the same, so the Ns can be deleted and duplicated sequence removed.

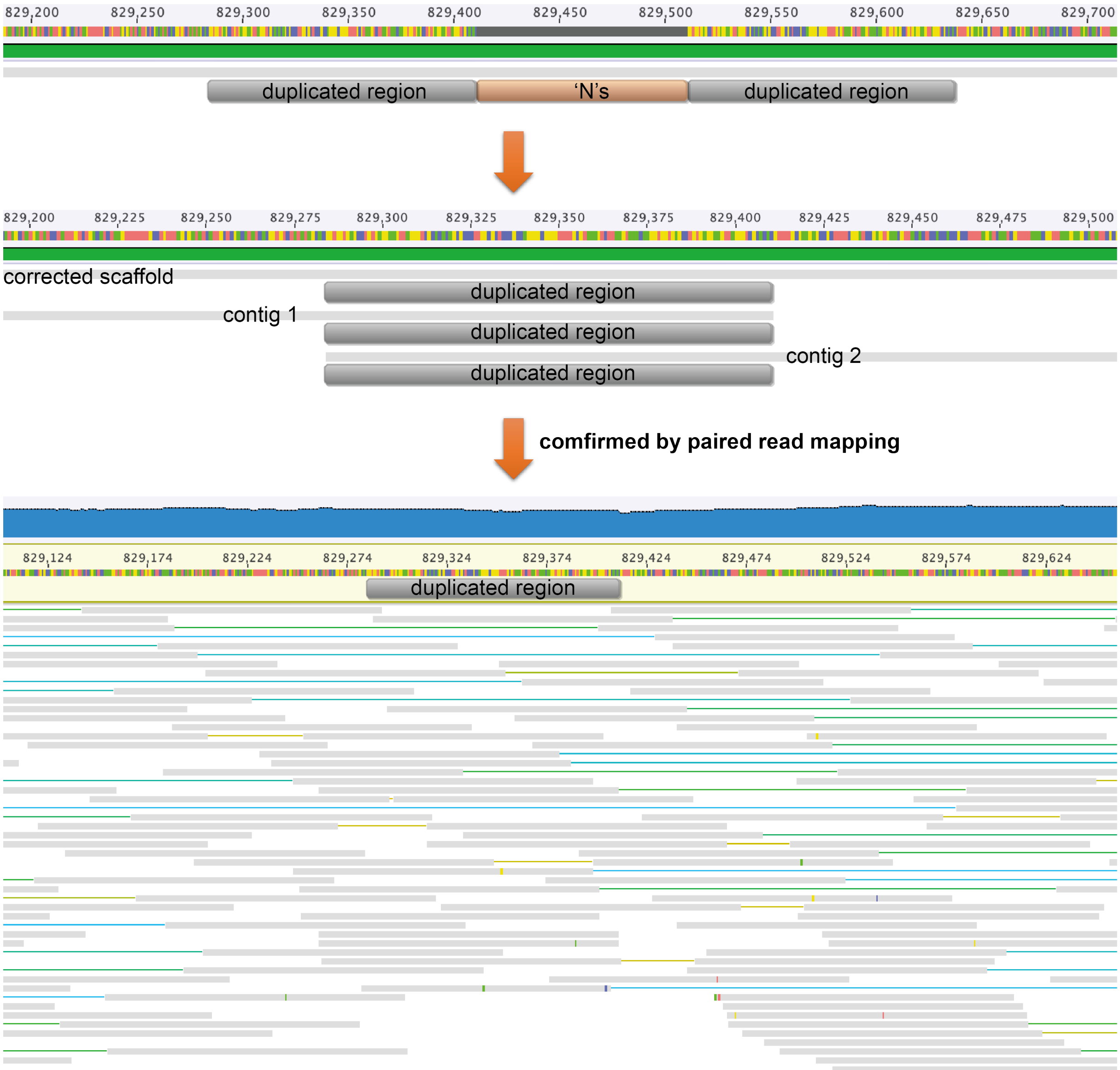

Supplemental Fig S4. The diagram shows a local error where Ns were wrongly inserted during the scaffolding step.
