# Supplementary material for: Accurate and complete genomes from metagenomes
Source: Genome Res. 2020 Mar;30(3):315–33. doi: 10.1101/gr.258640.119 (PMC7111523; doi:10.1101/gr.258640.119)
Supplement: Supplemental Material [file supp_gr.258640.119_Supplemental_Fig_S5.pdf]

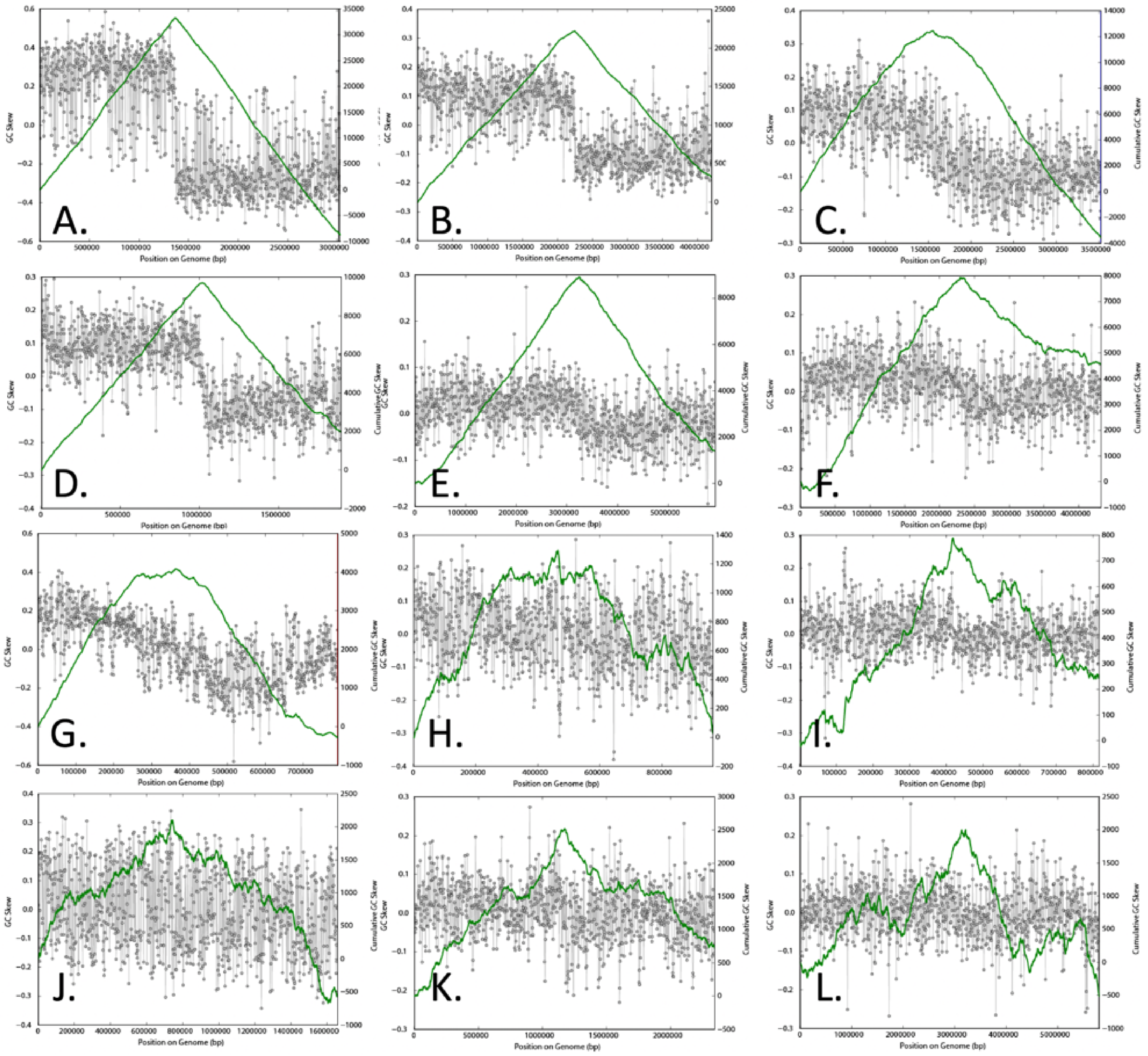


**Supplemental Fig S5.** Plots of GC skew and cumulative GC skew illustrating variations in patterns across 12 complete genomes for bacterial isolates. **A.** NC_003366.1, *Clostridium perfringens* str. 13, PUBMED 11792842, **B**. NC_002570.2, *Bacillus halodurans* C-125, PUBMED 10972189, **C.** NC_006138.1, *Desulfotalea psychrophila* LSv54, DOI: 10.1111/j.1462-2920.2004.00665x, **D.** NC_004606.1, *Streptococcus pyogenes* SSI-1, PUBMED 12799345, **E.** NC_005773.3, *Pseudomonas savastanoi* pv. phaseolicola 1448A, PUBMED 16159782, **F.** NC_007802.1, *Jannaschia* sp. CCS1, no publication, **G.** NC_007292.1, *Candidatus* Blochmannia pennsylvanicus str. BPEN, PUBMED 16077009, **H.** NC_002771.1, *Mycoplasma pulmonis* UAB CTIP, PUBMED 11353084, **I.** NC_000912.1, *Mycoplasma pneumoniae* M129, DOI: 10.1093/nar/24.22.4420, **J.** NC_005072.1, *Prochlorococcus marinus* MED4, PUBMED 12917642, **K.** NC_002950.2, *Porphyromonas gingivalis* W83, PUBMED 12949112, **L.** NC_009523.1, *Roseiflexus* sp. RS-1, no publication.
