# Supplementary material for: Accurate and complete genomes from metagenomes
Source: Genome Res. 2020 Mar;30(3):315–33. doi: 10.1101/gr.258640.119 (PMC7111523; doi:10.1101/gr.258640.119)
Supplement: Supplemental Material [file supp_gr.258640.119_Supplemental_Fig_S6.pdf]

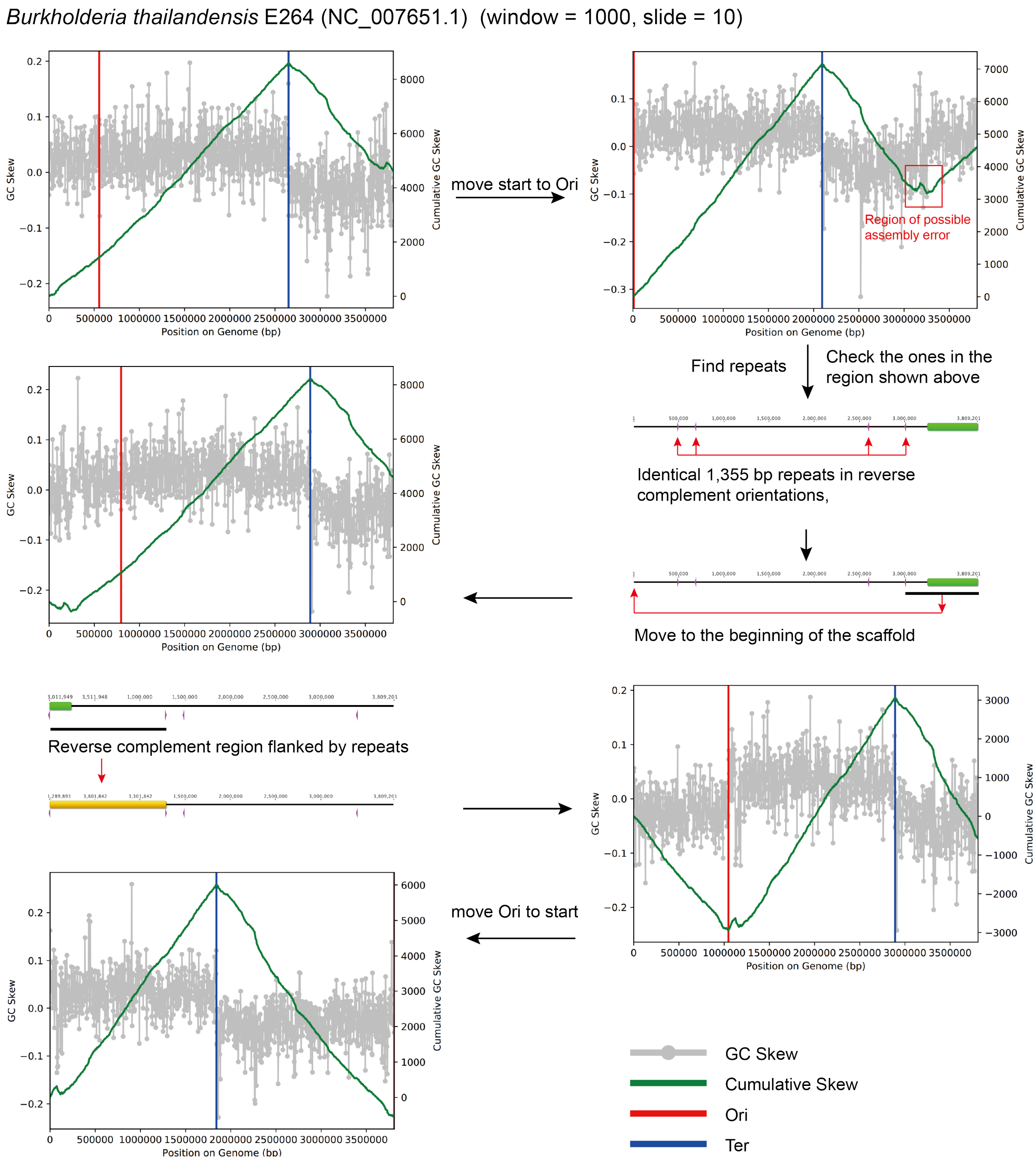

Supplemental Fig S6. The use of GC skew and repeat analysis to identify a possible assembly error in a RefSeq genome.
