# Supplementary material for: Accurate and complete genomes from metagenomes
Source: Genome Res. 2020 Mar;30(3):315–33. doi: 10.1101/gr.258640.119 (PMC7111523; doi:10.1101/gr.258640.119)
Supplement: Supplemental Material [file supp_gr.258640.119_Supplemental_Fig_S7.pdf]

(a) Acinetobacter baumanii (CP007712.1)

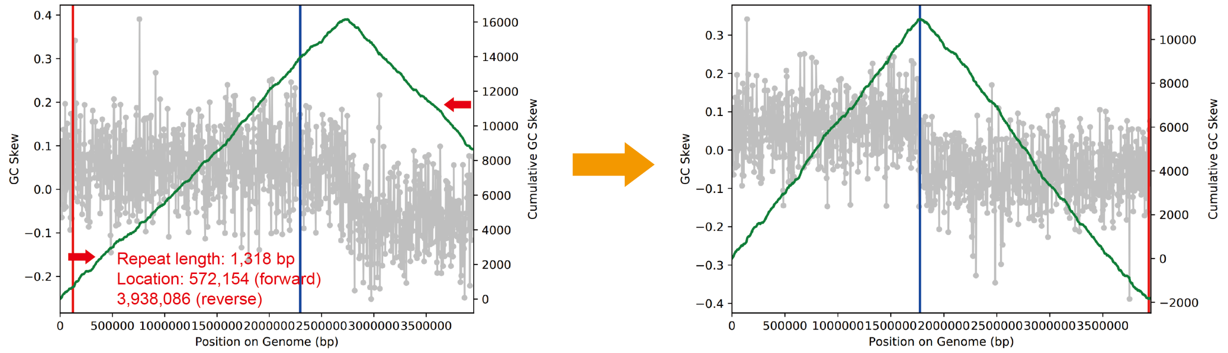

(b) Bacillus anthracis str. CDC 684 (NC_012581.1)

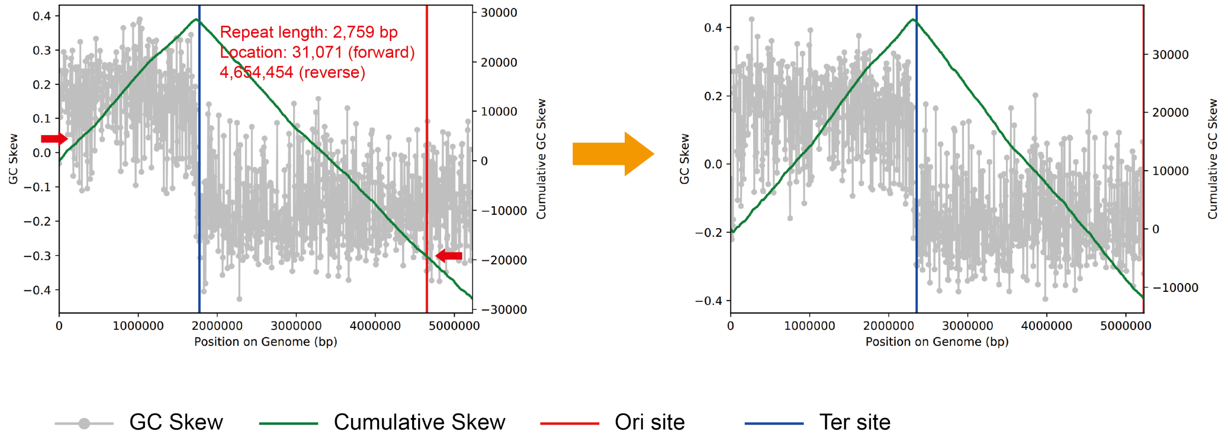

Supplemental Fig S7. Additional examples where abnormal patterns of GC skew and cumulative GC skew suggested the presence of assembly errors in RefSeq bacterial genomes. We identified repeats (likely longer than the distance spanned by paired reads) that were in reverse complemented orientations so the intervening sequence block could be flipped and the resulting GC skew pattern profiled.
