# Supplementary material for: Accurate and complete genomes from metagenomes
Source: Genome Res. 2020 Mar;30(3):315–33. doi: 10.1101/gr.258640.119 (PMC7111523; doi:10.1101/gr.258640.119)
Supplement: Supplemental Material [file supp_gr.258640.119_Supplemental_Fig_S8.pdf]

a

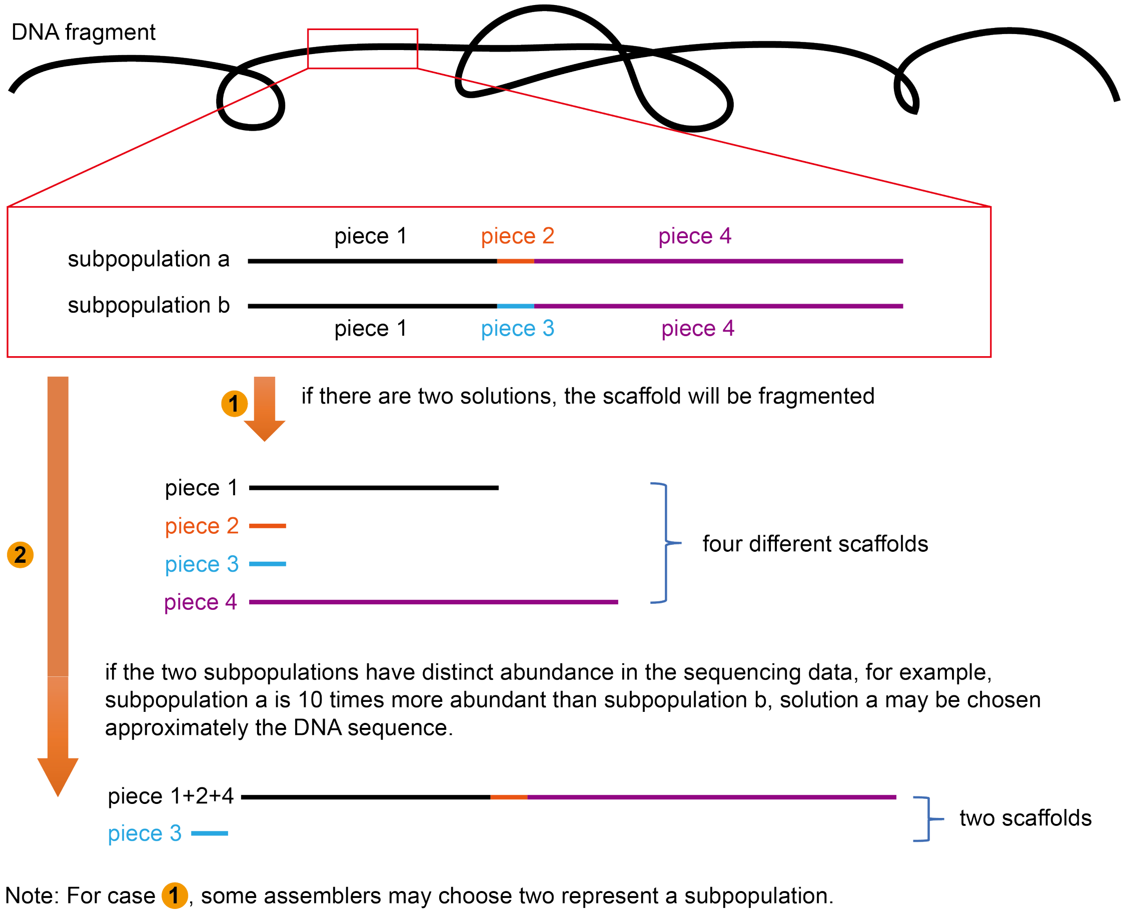

b

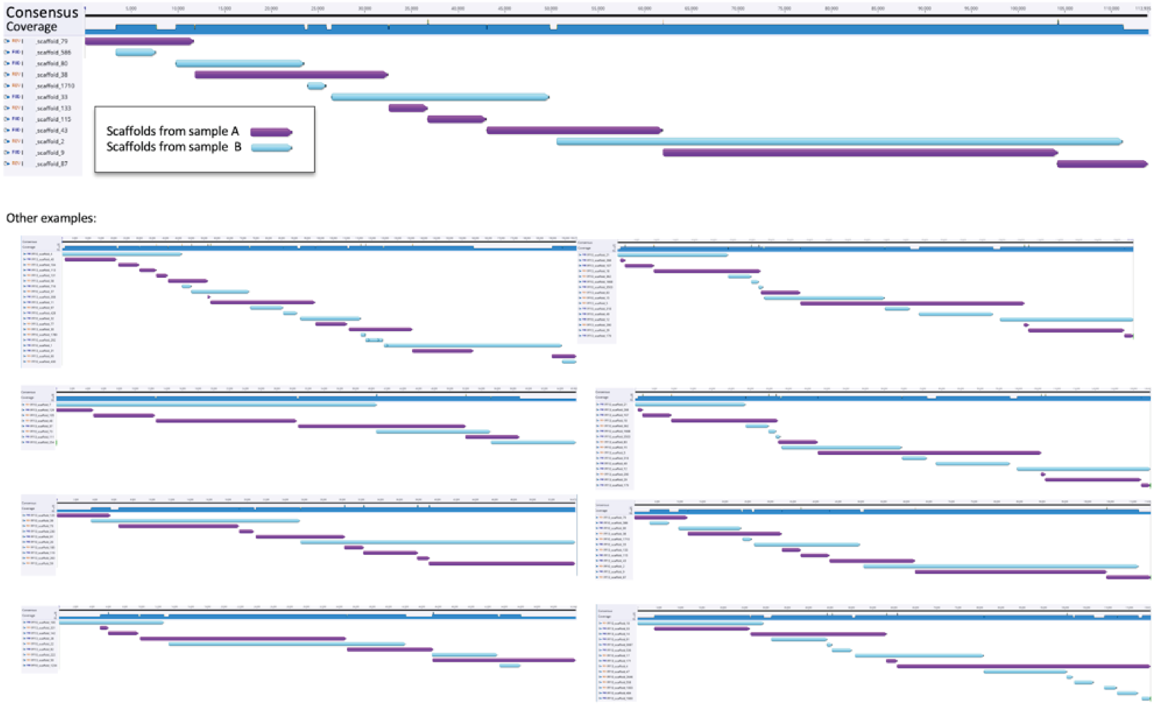

Supplemental Fig S8. (A) A possible solution when a scaffold breaks during assembly due to subpopulation variation. (B) In some cases, scaffold joins may be informed by the assembly of the same (or a very closely related) genome in another related sample (for example, from another time/depth/treatment). As shown below, both assembly paths are informed by the assembly of the same genome in another sample. Scaffold joins made based on data from another sample should be treated as hypotheses, and the entire genome verified by read mapping from a single sample. Different fragmentation patterns can occur due to differences in population structure between the samples or differences in coverage.
