# Supplementary material for: Accurate and complete genomes from metagenomes
Source: Genome Res. 2020 Mar;30(3):315–33. doi: 10.1101/gr.258640.119 (PMC7111523; doi:10.1101/gr.258640.119)
Supplement: Supplemental Material [file supp_gr.258640.119_Supplemental_Fig_S9.pdf]

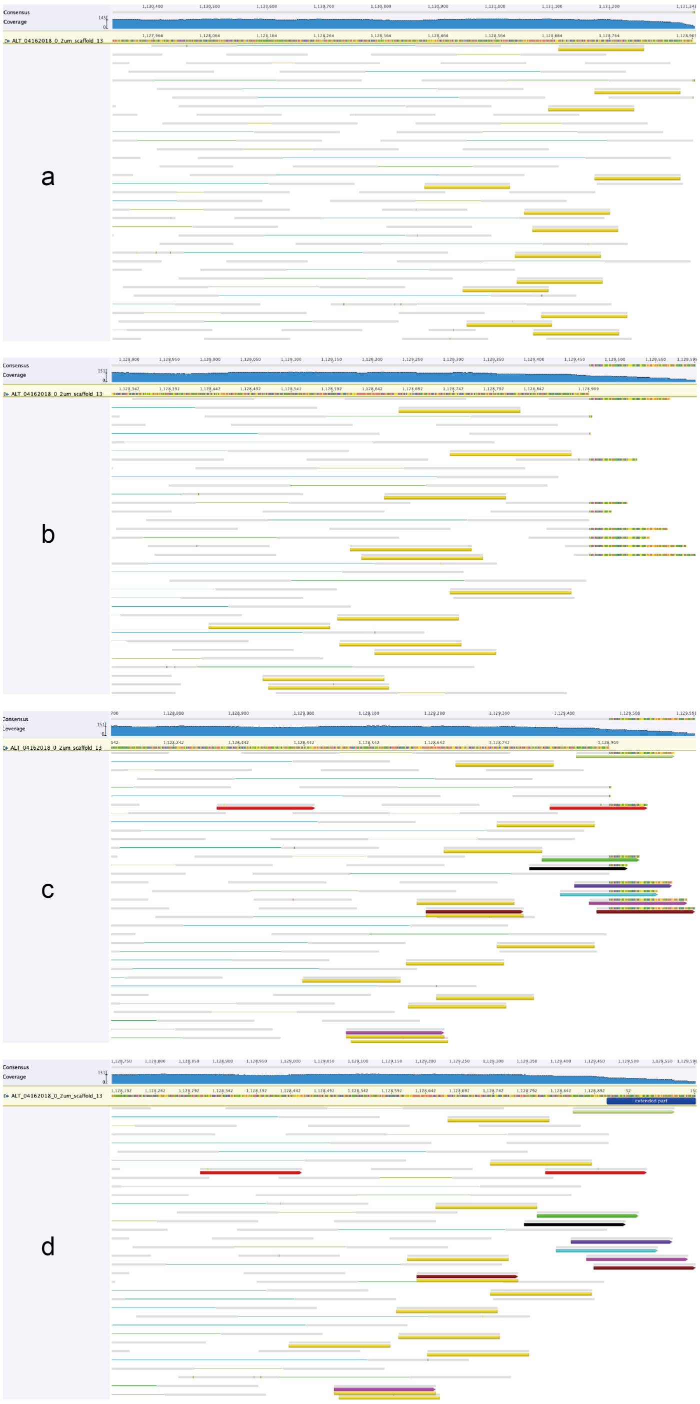

A subset of reads map to the end of the scaffold, but their mate pairs are unmapped.

The mapping of unplaced paired reads to the scaffold enables extension.

Colors indicate paired reads and show apporiate paired read separation.

The extended part of the scaffold based on consensus sequence of newly placed reads.

Supplemental Fig S9. The diagram shows the extension of the scaffold end by mapping paired-end reads to it. The extension could be performed for several cycles. Sometimes searching the extended sequence against the whole metagenome can bring in a missing fragment to substantially extend the scaffold. Joins are checked by read mapping. The figure showing the performance of scaffold extension using Geneious.
