# Supplementary material for: Accurate and complete genomes from metagenomes
Source: Genome Res. 2020 Mar;30(3):315–33. doi: 10.1101/gr.258640.119 (PMC7111523; doi:10.1101/gr.258640.119)
Supplement: Supplemental Material [file supp_gr.258640.119_Supplemental_Fig_S10.pdf]

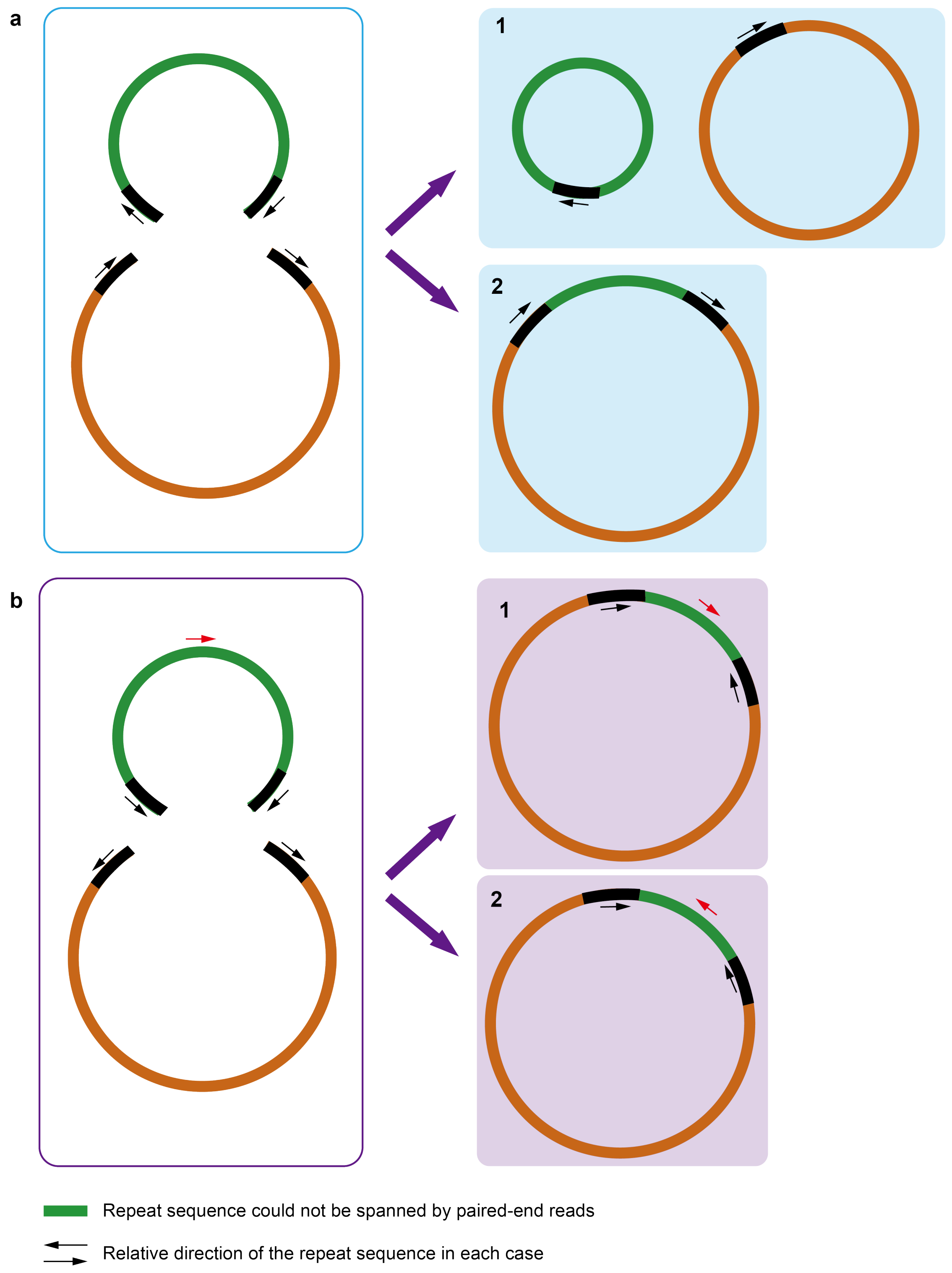

Supplemental Fig S10. Diagrams showing two possible solutions for two cases where a genome is in two pieces and there is no direct solution due to large repeat sequences that could not be spanned by paired-end reads. A. and B. differ in the orientations of the repeated sequences (see arrows). In case A, resolution into a single circular chromosome may be most likely. In the case of B, the options may be distinguished using GC skew information.
