# Supplementary material for: Accurate and complete genomes from metagenomes
Source: Genome Res. 2020 Mar;30(3):315–33. doi: 10.1101/gr.258640.119 (PMC7111523; doi:10.1101/gr.258640.119)
Supplement: Supplemental Material [file supp_gr.258640.119_Supplemental_Methods.pdf]

**Supplemental Methods**

**for**

**Accurate and Complete Genomes from Metagenomes**

Lin-Xing Chen[1], Karthik Anantharaman[1,7], Alon Shaiber[2,3], A. Murat Eren[3,4,]*, and Jillian F. Banfield[1,5,6]*

[1] Department of Earth and Planetary Sciences, Berkeley, CA, USA.
[2] Graduate Program in Biophysical Sciences, University of Chicago, Chicago, IL 60637, USA.
[3] Department of Medicine, University of Chicago, Chicago 60637 IL, USA.
[4] Bay Paul Center, Marine Biological Laboratory, Woods Hole 02543 MA, USA.
[5] Department of Environmental Science, Policy, and Management, Berkeley, CA, USA.
[6] Earth and Environmental Sciences, Lawrence Berkeley National Laboratory, Berkeley, CA, USA.
[7] Present address: Department of Bacteriology, University of Wisconsin, Madison, WI, USA.

*Corresponding authors:
Email: jbanfield@berkeley.edu
Telephone: 510-316-4334
Address: McCone Hall, Berkeley, CA 94720

Email: a.murat.eren@gmail.com
Telephone: 773-702-5935
Address: 900 E. 57th St., Chicago, IL 60637 USA

**Running title: Curated and complete metagenome-assembled genomes**

Below are the detailed descriptions of step-by-step procedures we performed to get complete or higher-quality genomes as illustrated as case studies in the main text.

***Case one, a CPR genome curated to completion***. In the step-by-step procedures shown below, we first obtained the mapped reads file for ALT_04162018_0_2um_scaffold_13 (hereafter referred to as "ALT_scaffold_13"). The graph of the coverage of paired reads mapped to ALT_scaffold_13 is shown below.

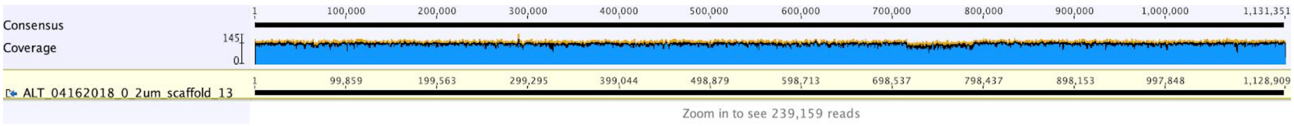


The ends of a scaffold could be extended as shown in Figure S6. After the extension of the scaffold, we searched the extended parts of against the whole metagenome scaffold set to retrieve fragments missed from the genome using BLASTn. Short pieces were identified that link the two ends of ALT_scaffold_13. However, the solutions were not unique due to the presence of SNVs. MetaSPades chose one path whereas IDBA_UD broke the assembly at this position.

***Confirmation of circularization***: The two ends of the scaffold shared the same sequence. We mapped reads to the scaffold (after trimming duplicated sequence) and confirmed the circularization by the detection of paired reads spanning the two ends (see below). Arrows underlining reads with the same color are paired reads (examples shown only).

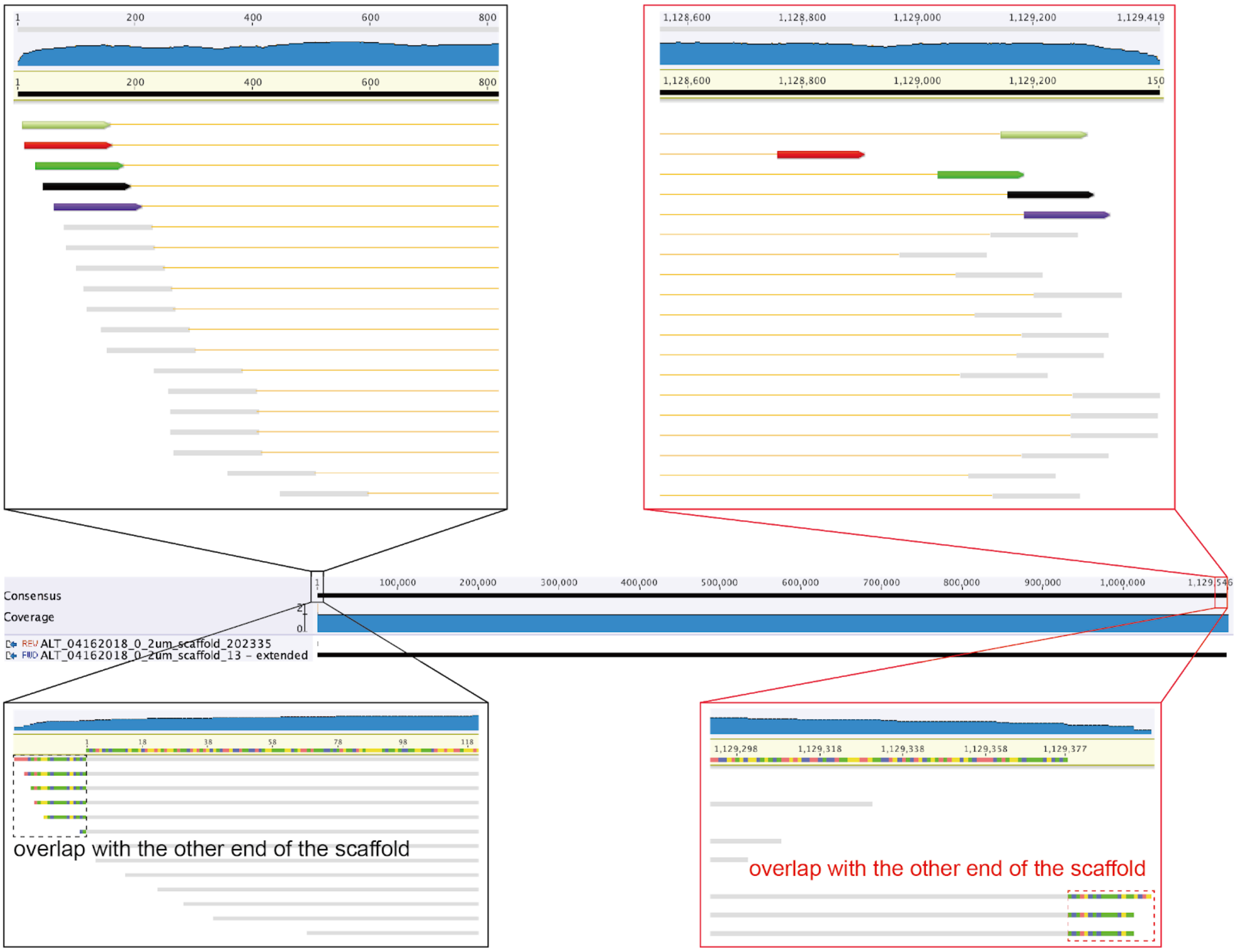

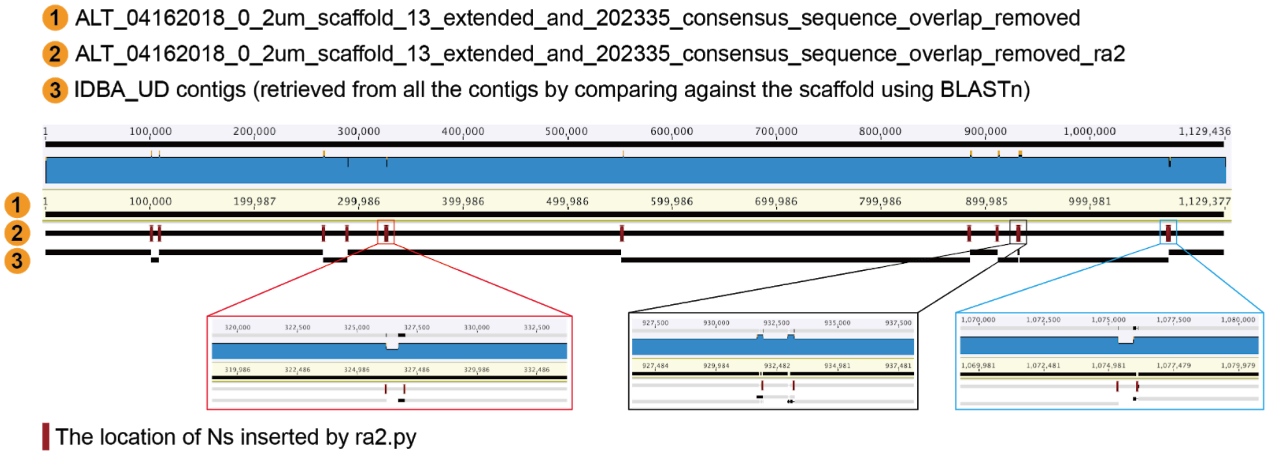


Diagram showing that local assembly errors often correspond to scaffolding joins made by the IDBA_UD assembler. 1. The scaffolds produced by IDBA_UD. 2. A total of 13 local assembly errors were reported by ra2.py (boxes). 3. The contigs generated by IDBA_UD (no scaffolding step) mapped to the scaffold. Three examples comparing the scaffolds and contigs are shown below. Errors that could not be fixed by ra2 were reported and curated manually. 12 of 13 local assembly errors were fixed as illustrated in Figures S3 and S4.

The thirteenth error is in a protein-coding gene that contains multiple repeats (see below). We used the coverage of the repeat region to approximate the repeat copy number, as the problem could not be solved directly.

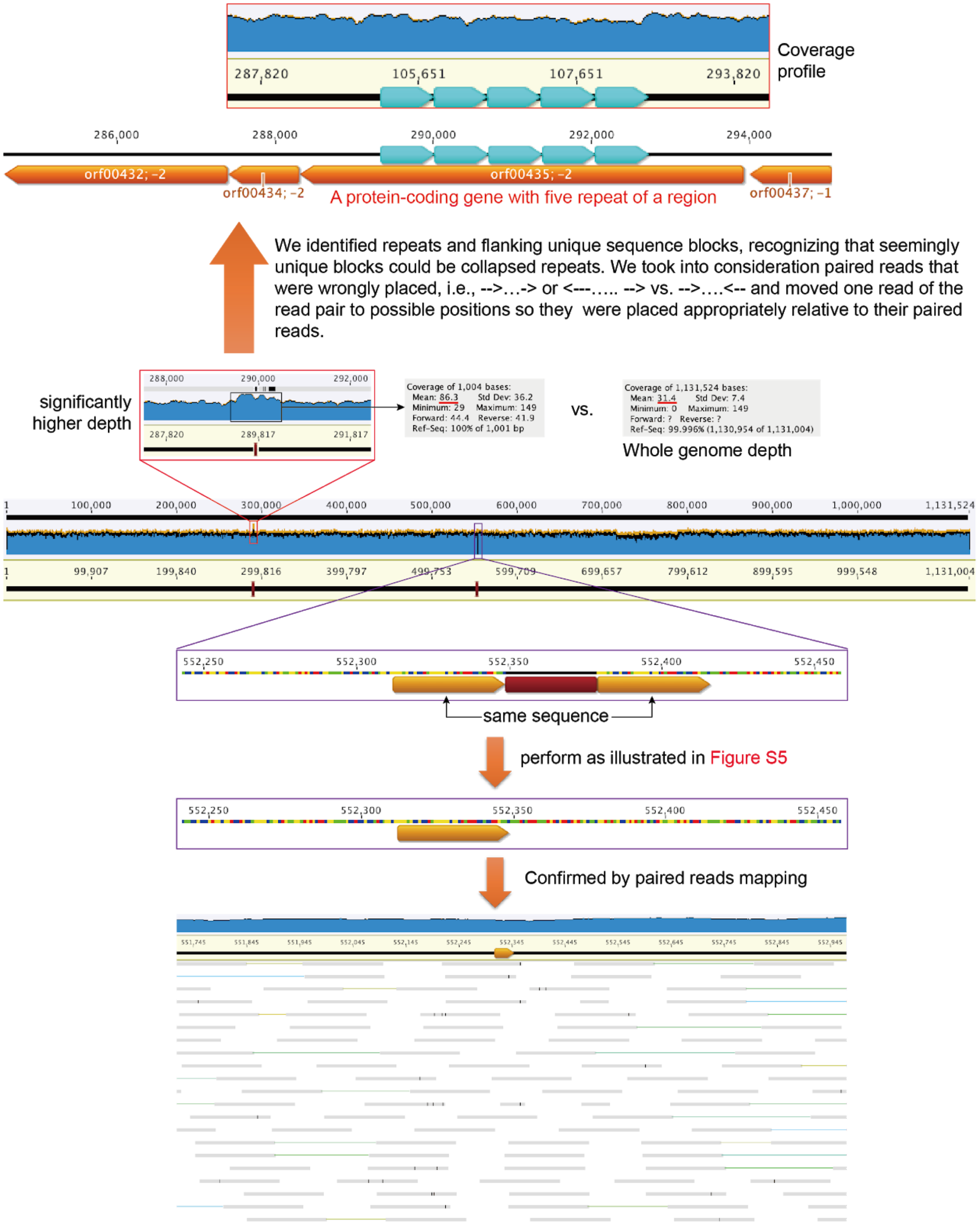

Coverage profile
287,820
105,651
107,651
293,820
286,000
288,000
290,000
292,000
294,000
orf00432; -2
orf00434; -2
orf00435; -2
orf00437; -1
A protein-coding gene with five repeat of a region
We identified repeats and flanking unique sequence blocks, recognizing that seemingly unique blocks could be collapsed repeats. We took into consideration paired reads that were wrongly placed, i.e., -->…-> or <---….. --> vs. -->….<-- and moved one read of the read pair to possible positions so they were placed appropriately relative to their paired reads.
significantly higher depth
Coverage of 1,004 bases:
Mean: 86.3 Std Dev: 36.2
Minimum: 29 Maximum: 149
Forward: 44.4 Reverse: 41.9
Ref-Seq: 100% of 1,001 bp
vs.
Coverage of 1,131,524 bases:
Mean: 31.4 Std Dev: 7.4
Minimum: 0 Maximum: 149
Forward: ? Reverse: ?
Ref-Seq: 99.996% (1,130,954 of 1,131,004)
Whole genome depth
same sequence
perform as illustrated in Figure S5
Confirmed by paired reads mapping

***Final check and adjust based on the origin of replication***: after all errors were fixed, paired reads were mapped to the genome with no mismatch allowed. As expected, all bases of the genome were well covered by reads and the coverage of the genome by reads was consistent throughout. One region had somewhat lower coverage due to the presence of prophage genome in a subset of cells. The GC skew and cumulative GC skew were calculated and the scaffold start changed to correspond with an intergenic region close to the location of the origin of replication.

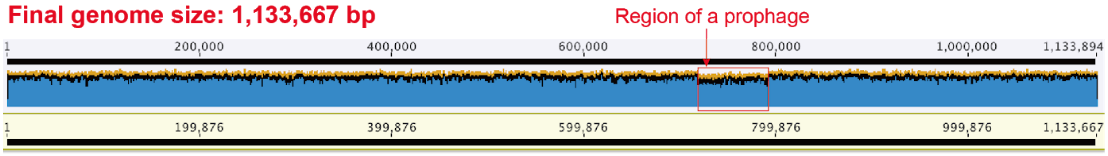


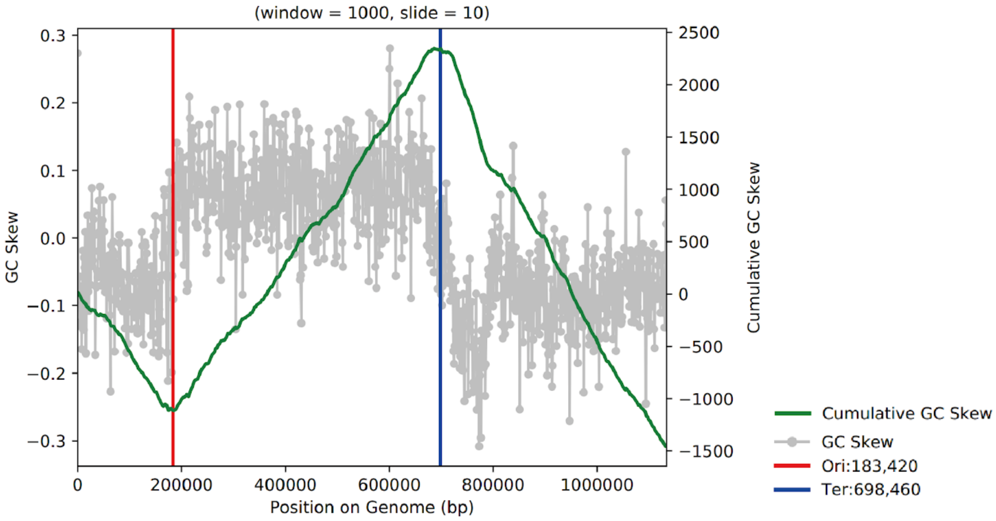


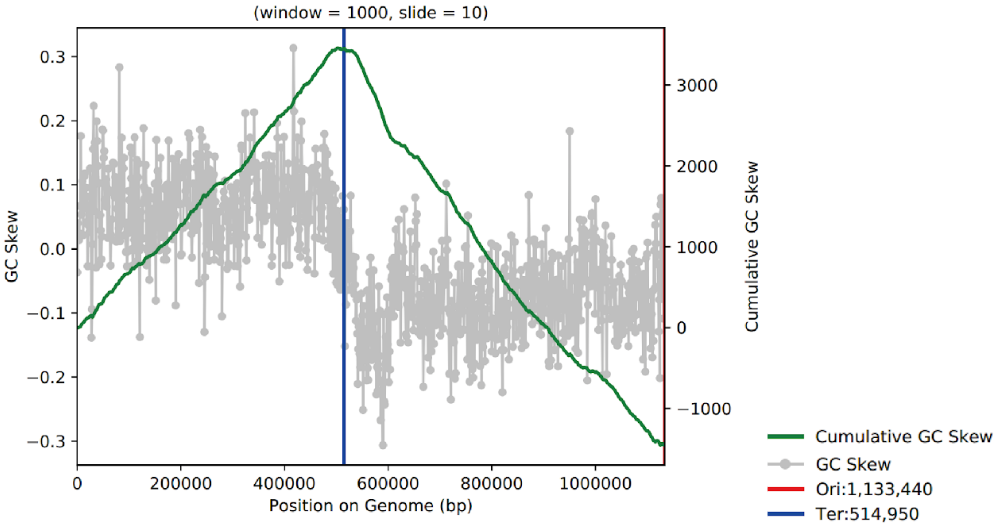

***Case two, curation of a Betaproteobacteria genome from a MAG comprised of 7 scaffolds.***

The scaffolds were extended for the 1st run (see the extended part in blue in Figure below).

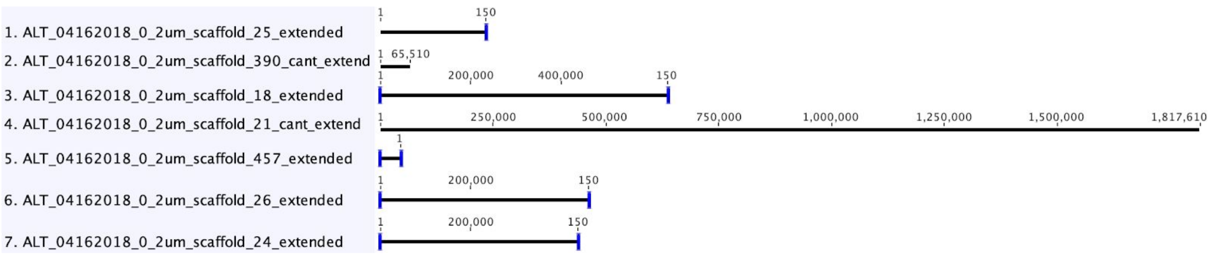


Two of the scaffolds (scaffold_21 and scaffold_25) could be assembled based on overlap and confirmed by read mapping (see below).

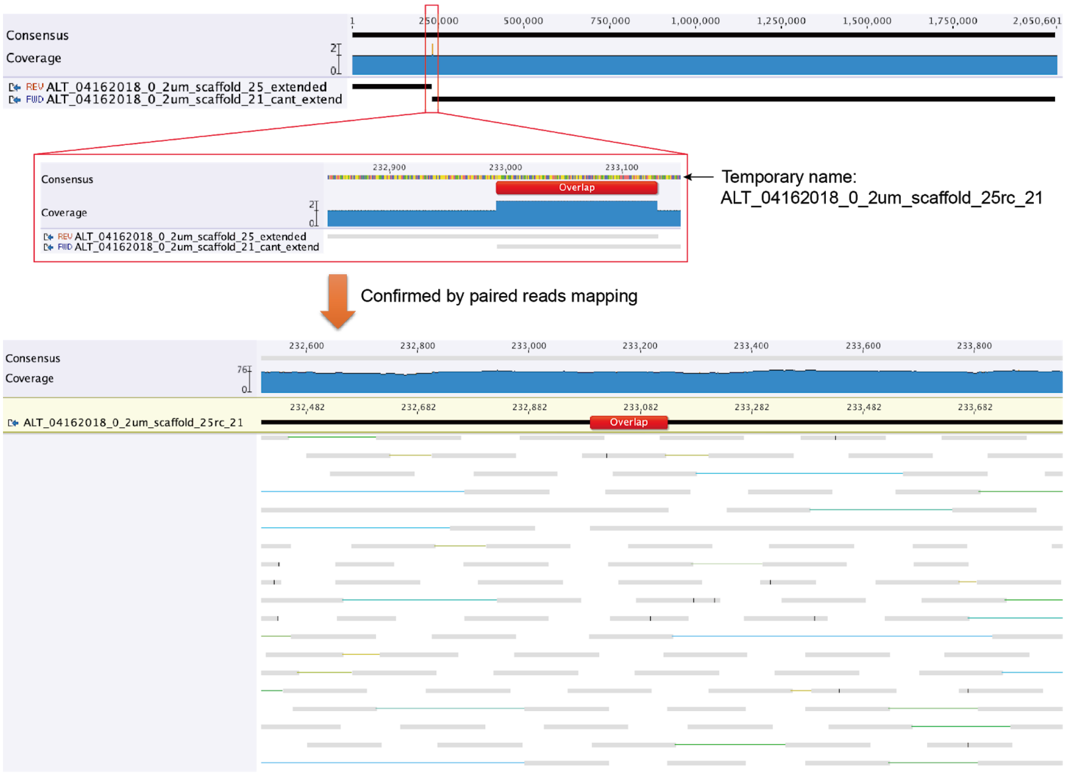


The linkage of scaffolds in the bin using fragments from the whole metagenome. Linking scaffolds were identified by BLASTn of newly extended parts of scaffolds to the full data. Possible linkages were established using "overlap-based assembly" and confirmed by reads mapping. We also considered constraints provided by the *Sulfuricella denitrificans* skB26 genome. When all the scaffolds were combined into two large genome fragments, there were two choices of how they could be arrayed. One choice, "assembled_scaffold_1 + assembled_scaffold_2" has the expected GC skew and is likely the correct solution.

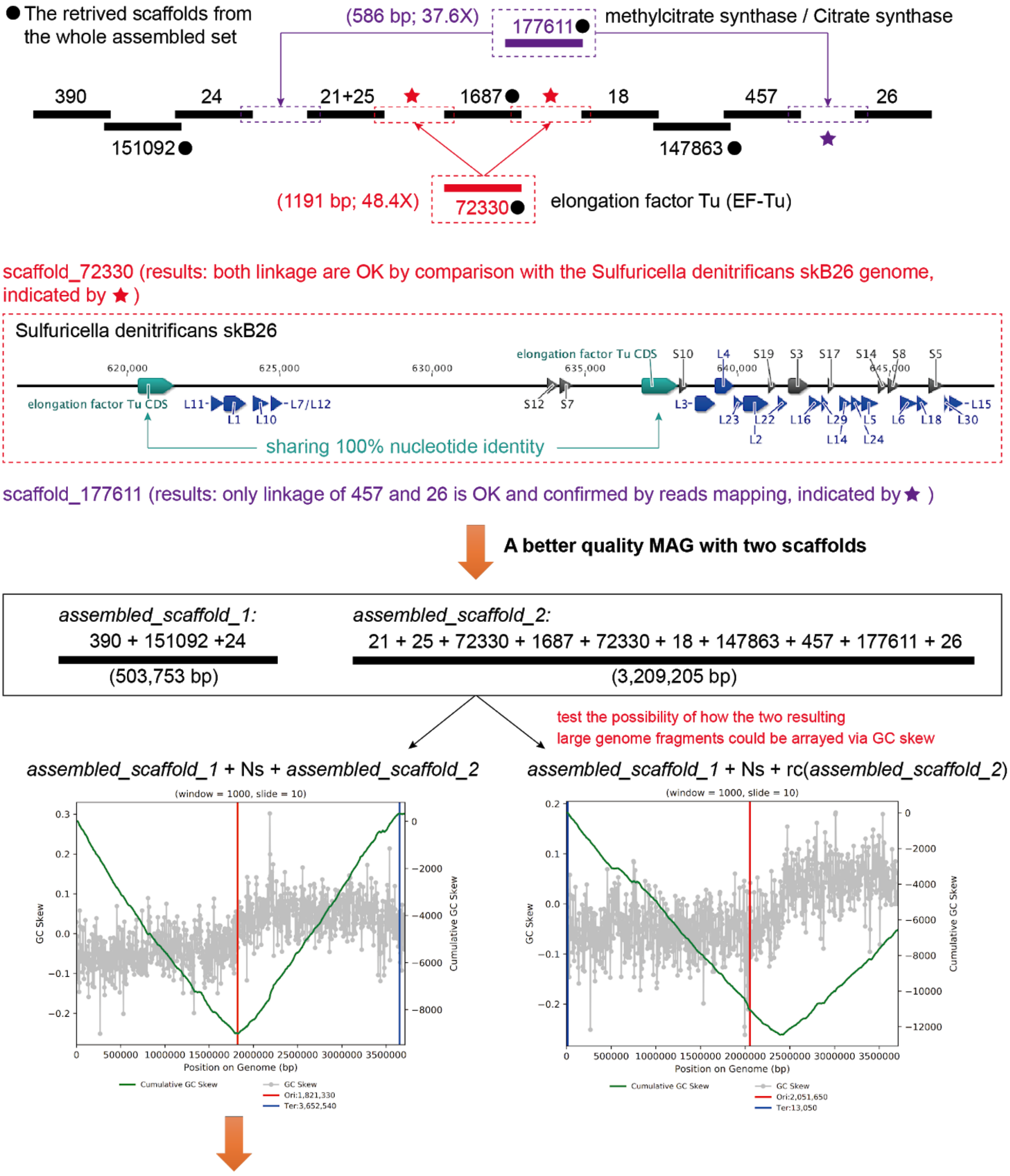


*assembled_scaffold_1* + Ns + *assembled_scaffold_2* represents the likely correct linkage of the resulting large genome fragment, the GC skew patten indicated that the genome is near complete after curation.

**Case three.** the curation of a published incomplete genome to completion was achieved by filling two closely spaced gaps. In this diagram, bars of the same length and color have the same sequence.

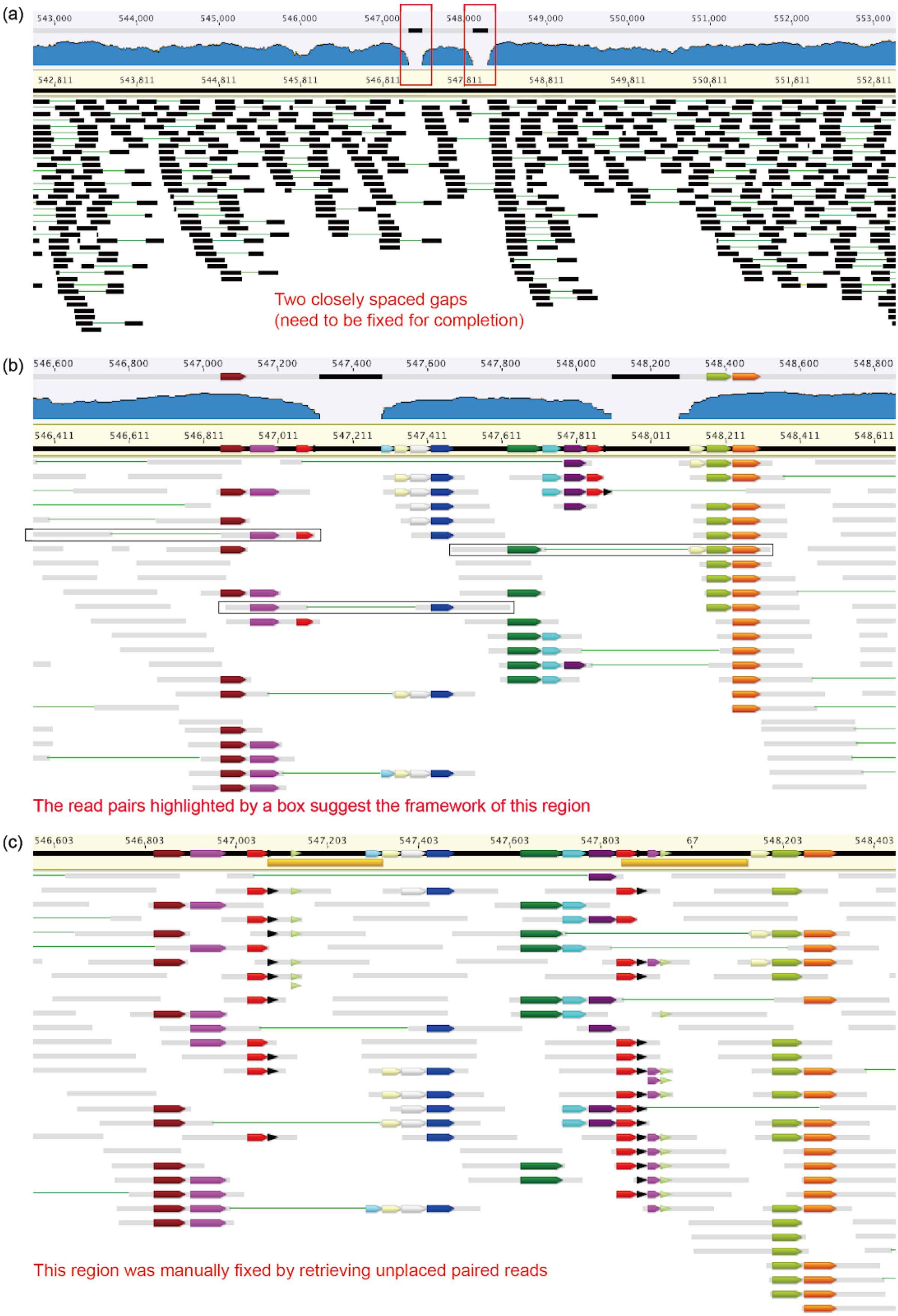

**Scaffold Extension and Gap Closing**

A blog by Lin-Xing Chen, Karthik Anantharaman, Alon Shaiber, A. Murat Eren, and Jillian F. Banfield.

**Why?**

This workflow describes the detailed step-by-step procedures for scaffold extension and gap closing as described in the paper entitled “Accurate and Complete Genomes from Metagenomes” ("CGM" for short hereafter), the preprint is available at https://doi.org/10.1101/808410.

The CGM paper summarizes the history of genome-resolved metagenomics, with examples from publications, to show the necessity of genome binning and manual curation of binned genomes (or metagenome-assembled genomes; MAGs) to avoid misleading conclusions. The paper provided a step-by-step procedure of how to curate a draft MAG generated by manual or automatic binning tools to prevent errors (e.g., misbinned scaffolds, local assembly errors), close scaffolding gaps and thus generate high-quality or complete MAGs (CMAGs). Here, we provide a more detailed procedure.

**What is “scaffold extension”?**

Scaffold extension uses the paired-end reads mapped to a given scaffold to extend its ends, (1) to get the complete sequence of a protein-coding gene or rRNA gene at the ends, (2) to assemble it with another scaffold based on overlap sequences at the end, (3) to obtain a complete genome of bacteria, archaea, virus, phage, etc.

**In the CGM paper, we wrote**:

“Scaffolds within a bin that do not overlap at the start of curation may be joined after one or more rounds of scaffold extension. This process of extending, joining and remapping may continue until all fragments comprise a single circularized sequence. It should be noted that read by read scaffold extension is very time-consuming. If an extended scaffold cannot be joined to another scaffold after a few rounds of extension it may be worth testing for an additional scaffold (possibly small, thus easily missed by binning) by searching the full metagenome for overlaps. Sometimes, the failure of scaffold extension is due to missing paired reads, which may be found at the end of another fragment. If they are pointing out but the sequences cannot be joined based on end overlap, a scaffolding gap can be inserted in the joined sequence (reverse complementing one of the scaffolds may be necessary).”

- which means that sometimes it is possible to link all the scaffolds of a draft MAG into a circular genome by scaffold extension followed by overlap-based assembly of the extended scaffolds. However, it should be noted that a manual scaffold extension is time-consuming and often does not lead to a circular MAG genome sequence. Additional tests should be performed to verify the accuracy of the final product (see CGM).

**How can scaffold extension be performed?**

In the CGM paper, we manually extended the scaffolds in a given bin to be curated using Geneious. There are several steps for this, including (1) mapping of reads to the scaffolds that comprise a bin, (2) visualization in Geneious and (3) manual extension in Geneious. Note that the reads used for extension should be appropriately placed relative to their already mapped pair, in the right orientation, and the originally mapped pair should support the consensus sequence at the scaffold end (see below for details).

**(1) Read mapping**

Read mapping can be performed with available tools, for example, bowtie2. Here, we illustrate using the first genome bin (comprising a single scaffold) described in the section of "Case studies illustrating the curation of draft MAGs" in CGM, i.e., ALT_04162018_0_2um_scaffold_13. The scaffold was assembled from paired-end reads (ALT_04162018_0_2um.1.fastq.gz and ALT_04162018_0_2um.2.fastq.gz) and in fasta format. Two steps are needed for read mapping using bowtie2.

First, build the database for mapping.

```
bowtie2-build ALT_04162018_0_2um_scaffold_13.fasta ALT_04162018_0_2um_scaffold_13.fasta
```

Second, map the reads to the scaffold

```
bowtie2 -p 6 -X 2000 -x ALT_04162018_0_2um_scaffold_13.fasta -1 ALT_04162018_0_2um.1.fastq.gz -2
ALT_04162018_0_2um.2.fastq.gz | shrinksam -v > ALT_04162018_0_2um_scaffold_13.fasta.mapped.sam
```

Note: "-p 6" is the number of cores used for mapping, "-X 2000" is the largest insert length allowed when mapping paired-end reads, which could be modified based on the study. `Shrinksam` is a tool to filter the original sam file from bowtie2, which will only write the mapped reads to the file and thus save a lot of storage room. `Shrinksam` could be downloaded from Github (https://github.com/bcthomas/shrinksam).

**(2) Visualization in Geneious**

Prepare the scaffold file (.fasta) and the bowtie2 mapping file (.sam), using the function in Geneious, "File" -> "Import" -> "From File".

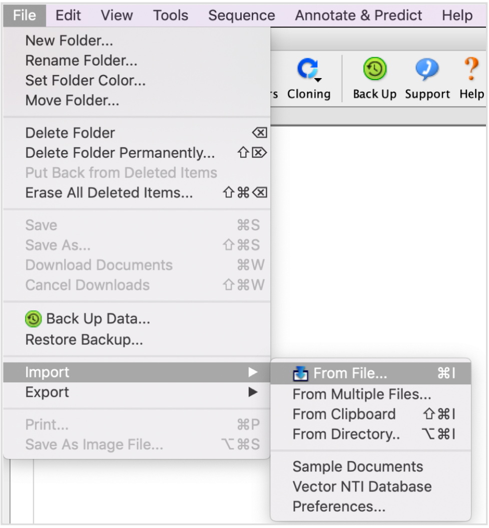


Once the fasta file is imported, select it and import the sam file.

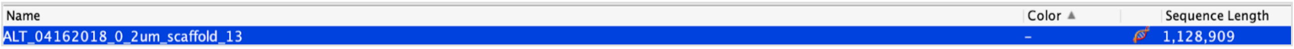


Select the appropriate .fasta sequence used for mapping:

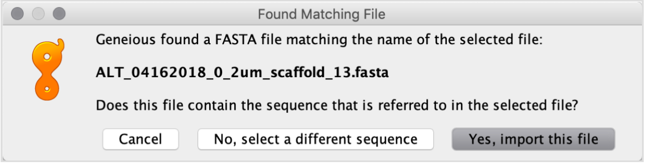


Two files are generated, one (the middle one in the screenshot below) shows the reads that are mapped to the scaffold, the other (the bottom one) includes the unplaced reads from pairs (means the other reads in those pairs were mapped to the scaffold via bowtie2).

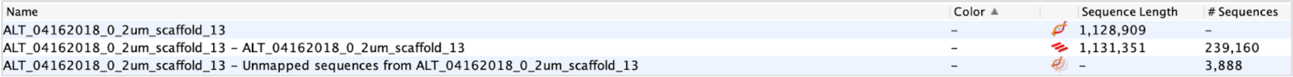


Below is the overview of all reads mapped to the scaffold, the coverage profile is generally even (excepting a region between 700,000 and 800,000 bp, which is a prophage region).

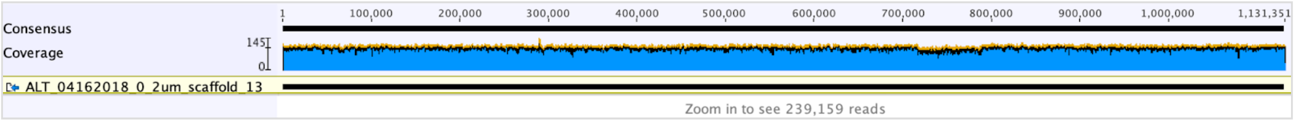


**(3) Manual extension of scaffold**

The next step is to extend the scaffold manually in Geneious. Firstly, select the fasta file and the unplace reads file (mentioned above), and use the Geneious function "Align/Assemble" -> "Map to Reference".

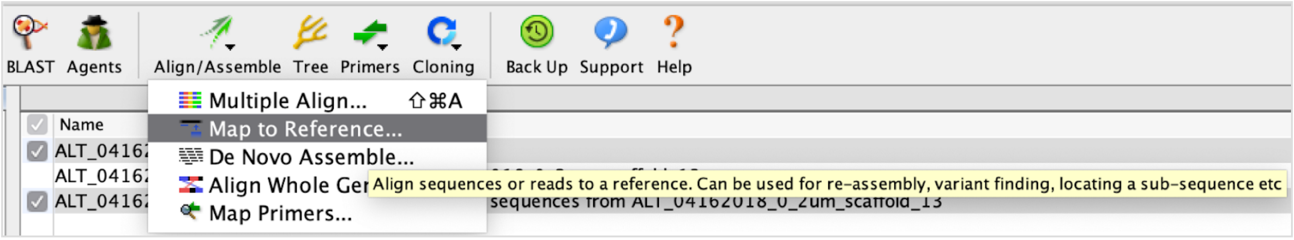


For sensitivity, use "Custom Sensitivity" and set “Maximum Mismatches Per Read” as 2%, do not change any other parameters. You can check "Save list of unused reads" so that the saved reads will be used for the next run of extension.

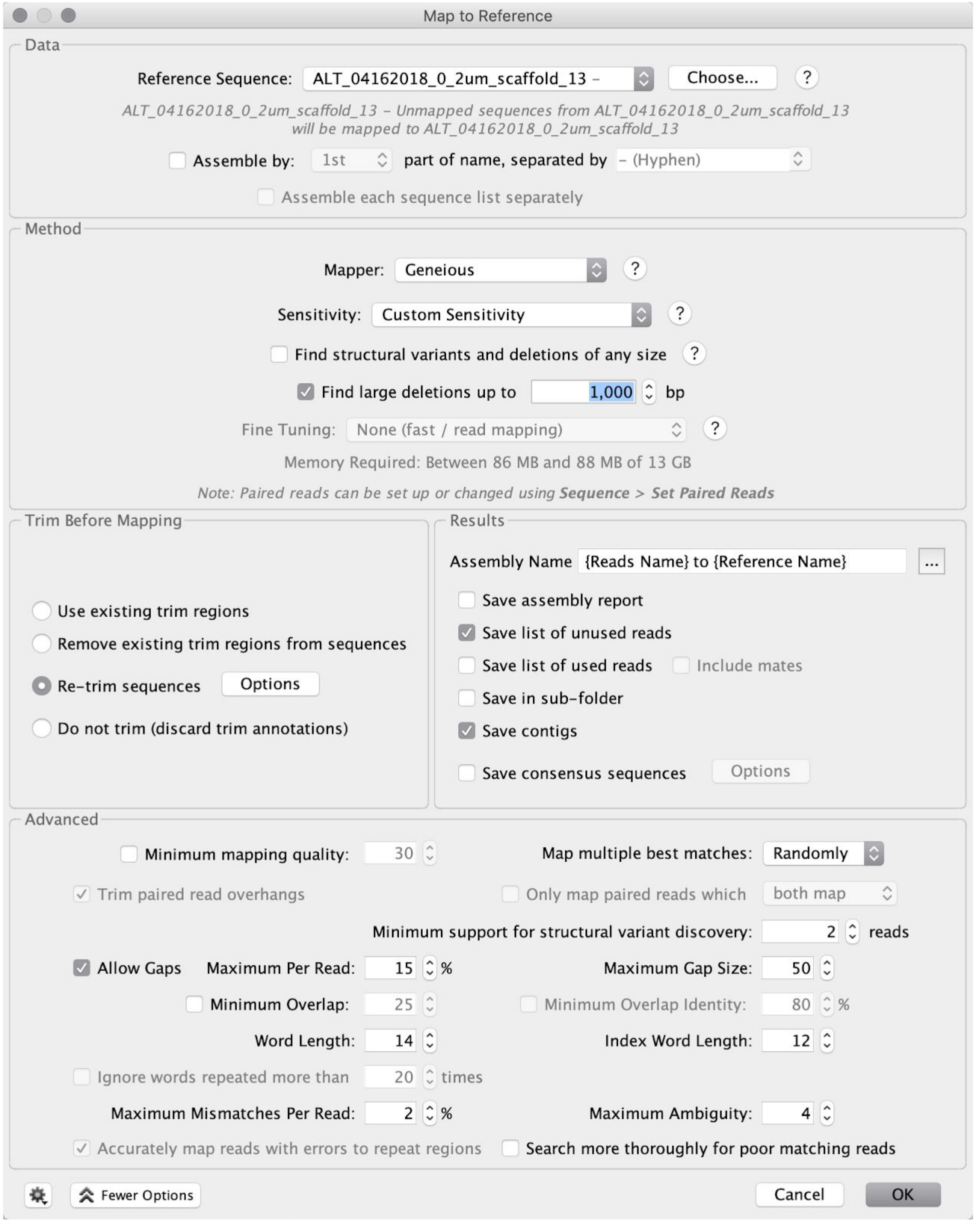


Two files are generated by this step (at the bottom of the screenshot below), with 605 unplaced reads from bowtie2 mapping mapped to the scaffold, and 3,284 still unmapped.

| Name | Color ▲ | Sequence Length | # Sequences |
|---|---|---|---|
| ALT_04162018_0_2um_scaffold_13 | – | 1,128,909 | – |
| ALT_04162018_0_2um_scaffold_13 – ALT_04162018_0_2um_scaffold_13 | – | 1,131,351 | 239,160 |
| ALT_04162018_0_2um_scaffold_13 – Unmapped sequences from ALT_04162018_0_2um_scaffold_13 | – | – | 3,888 |
| ALT_04162018_0_2um_scaffold_13 – Unmapped sequences from ALT_04162018_0_2um_scaffold_13 to ALT_04162018_0_2um_scaffold_13 | – | 1,129,382 | 605 |
| ALT_04162018_0_2um_scaffold_13 – Unmapped sequences from ALT_04162018_0_2um_scaffold_13 to ALT_04162018_0_2um_scaffold_13 Unused Reads | – | – | 3,284 |

Check the reads mapped to the ends of the scaffold. The consensus sequences of reads that only partially mapped to the scaffold should be used for extension (see Left and Right ends, below: copy and paste the read sequence into the consensus sequence line above them).

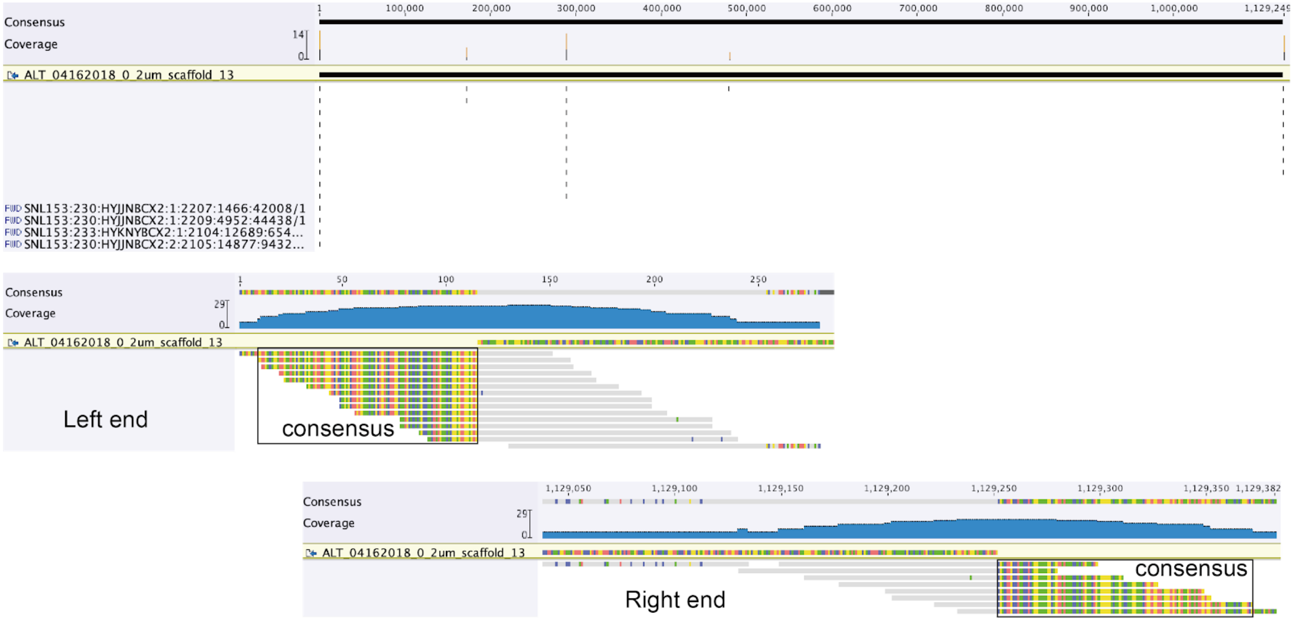


Before extending the scaffold, the reads in the pairs that already mapped to the scaffold should be checked to see if they are placed at an appropriate distance away, given the library size information. This can be done by searching the names of the reads. For example, the name of the most bottom read on the right end is "SNL153:233:HYKNYBCX2:1:1109:2879:14634/1 (reversed)", which could be copied as shown in the screenshot below.

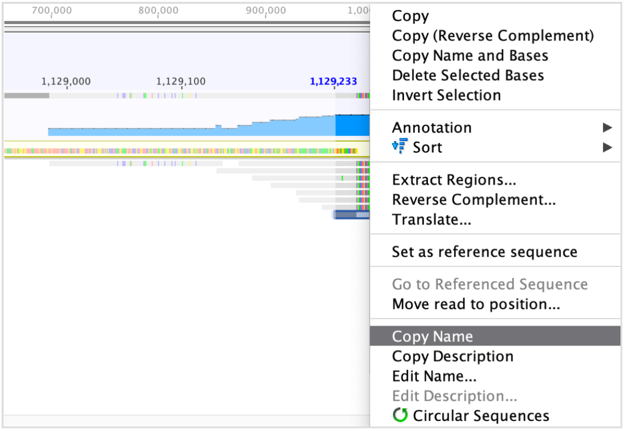


Thus, we should search "SNL153:233:HYKNYBCX2:1:1109:2879:14634/2" in the original sam file from bowtie2 to check its mapping location and orientation (see below).

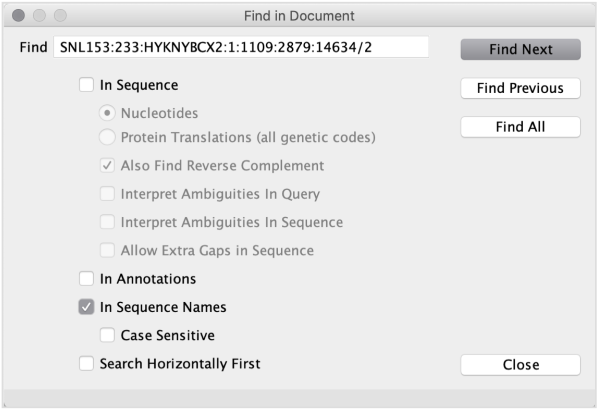

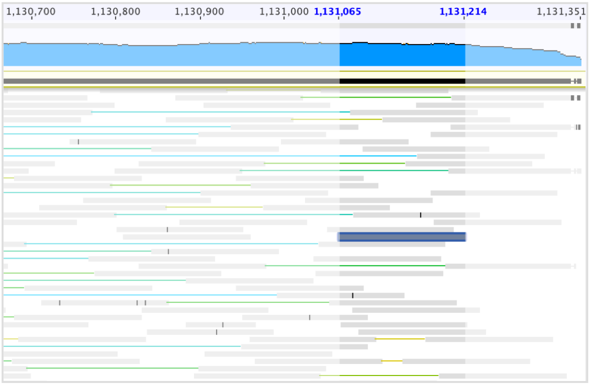


The extended  scaffold sequence can be saved as "ALT_04162018_0_2um_scaffold_13_extended" (from 1,128,909 bp to 1,129,134 bp in length).

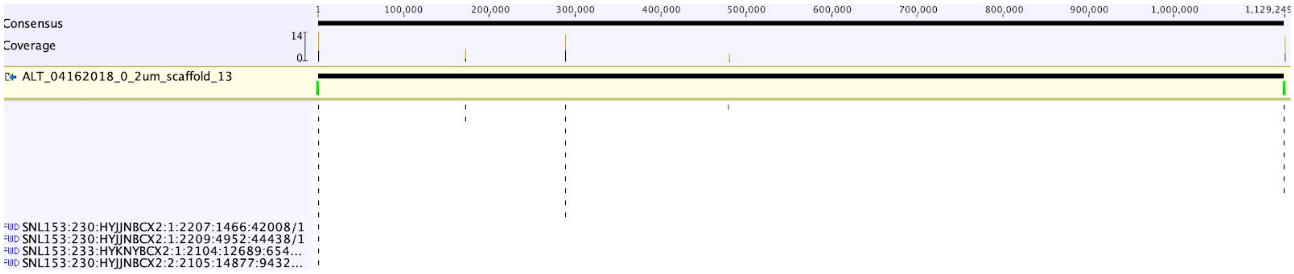


| Name | Color | Sequence Length | # Sequences |
|---|---|---|---|
| ALT_04162018_0_2um_scaffold_13 | - | 1,128,909 | - |
| ALT_04162018_0_2um_scaffold_13_extended | - | 1,129,134 | - |
| ALT_04162018_0_2um_scaffold_13 - ALT_04162018_0_2um_scaffold_13 | - | 1,131,351 | 239,160 |
| ALT_04162018_0_2um_scaffold_13 - Unmapped sequences from ALT_04162018_0_2um_scaffold_13 | - | - | 3,888 |
| ALT_04162018_0_2um_scaffold_13 - Unmapped sequences from ALT_04162018_0_2um_scaffold_13 to ALT_04162018_0_2um_scaffold_13 | - | 1,129,382 | 605 |
| ALT_04162018_0_2um_scaffold_13 - Unmapped sequences from ALT_04162018_0_2um_scaffold_13 to ALT_04162018_0_2um_scaffold_13 Unused Reads | - | - | 3,284 |

Then, map the unused reads set from the last mapping run to the extended scaffold. This should be repeated until no more unplaced reads are recruited. If necessary, the full metagenome read dataset can be remapped to  the extended scaffold sequence to continue the process.

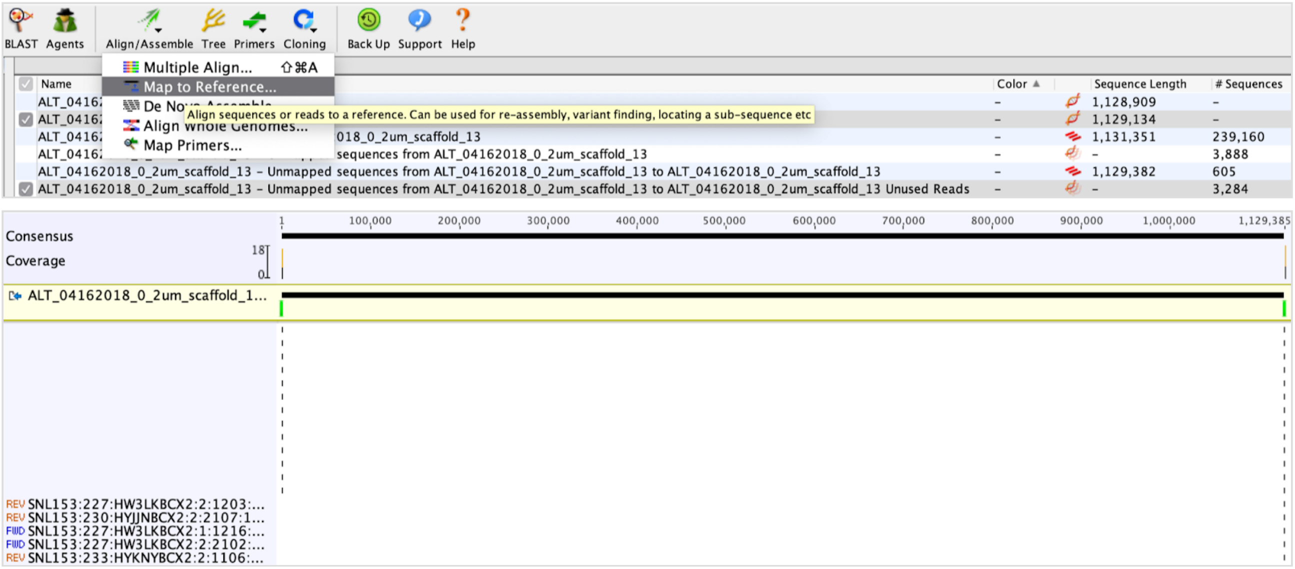


Often, we will see variations in the reads mapped to scaffold ends. These may indicate distinct paths or sequence variants (possibly subpopulations). As above, in some cases, it may be possible to determine the correct path because only one of two variants has a paired read that supports the consensus. If this isn't the case, some curators may

choose to follow the variant path with the most appropriate coverage (or the majority of reads), others may terminate the extension effort.

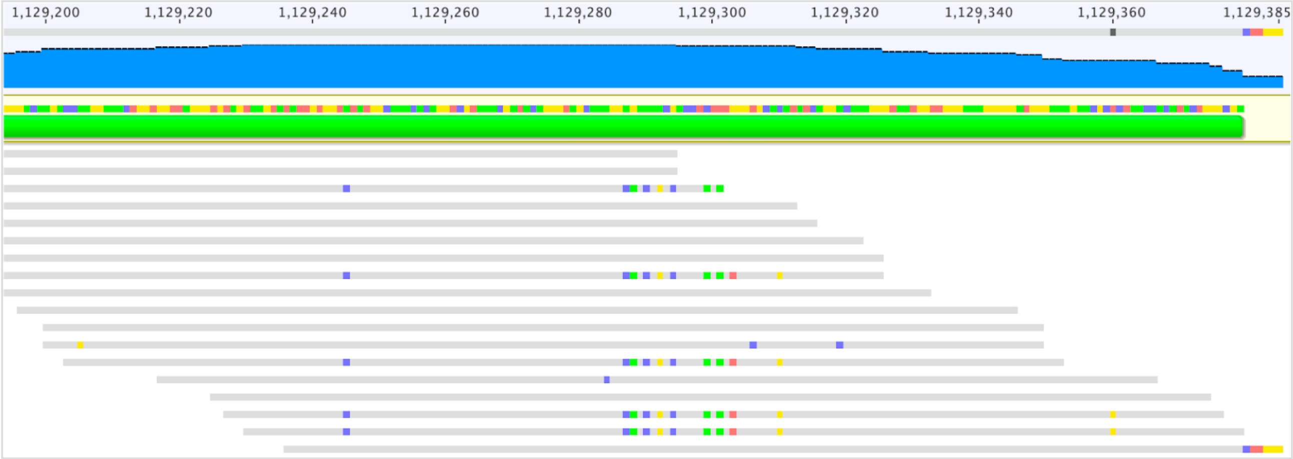


After a round of scaffold extension it may be possible to find scaffolds in the genome bin that can be joined (in some cases, spanned by paired reads). If no scaffold is found, the sequence of the new scaffold end can be searched against the entire metagenome to find a fragment that was not included in the bin (with the right GC content, coverage and phylogenetic profile). This sequence can be used to extend the scaffold, and reads remapped for further validation.

**What is gap closing?**

When scaffolding is a step of *de novo* assembly (assemblers like IDBA_UD, metaSPAdes) of a given metagenomic dataset, Ns are inserted between contigs spanned by paired-end reads. CMAGs represent genomes without Ns, thus the N gaps in the scaffolds should filled by the appropriate sequence

**How can gap closing be performed?**

The first step should be to check whether the N gap is indeed spanned by paired reads (if not, the assembly may be chimeric). The second step should be to see whether the sequence in the gap region is already present but duplicated on either side of the Ns (thus, the gap can be closed by removing the duplicated region and the Ns).

Gap closing can be performed by automatic tools like Gapfiller (Nadalin et al. 2012) but the performance has not been evaluated by us. Here, we describe how this can be done in Geneious.

To illustrate, we use another scaffold from the same sample as the one we described above, i.e., ALT_04162018_0_2um_scaffold_30, which contains a scaffolding gap (these gaps can be quickly located by searching "Ns").

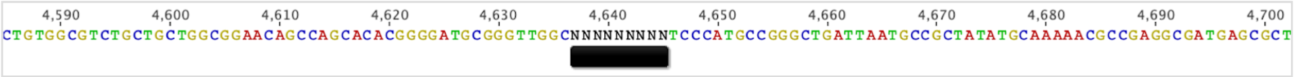


This is how the read mapping profiles from bowtie2 look like.

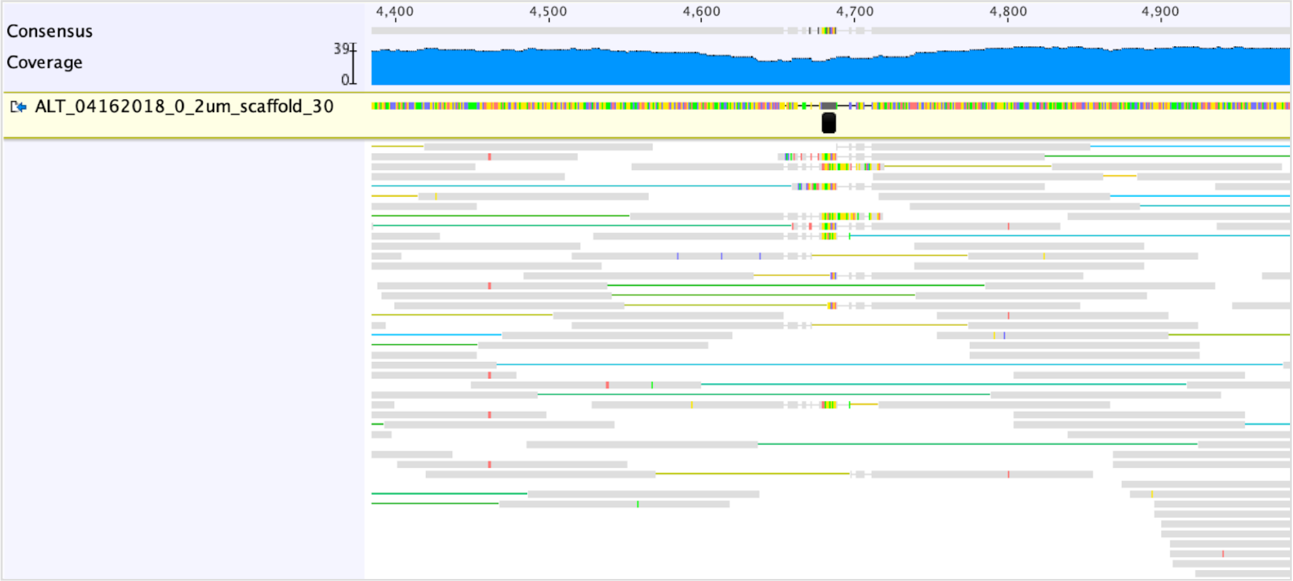

The first step to fix the scaffolding gap is to open the gap, by adding a suitable number of "-" into the Consensus sequence (the grey line in the screenshot below shows the right place to add the "-").

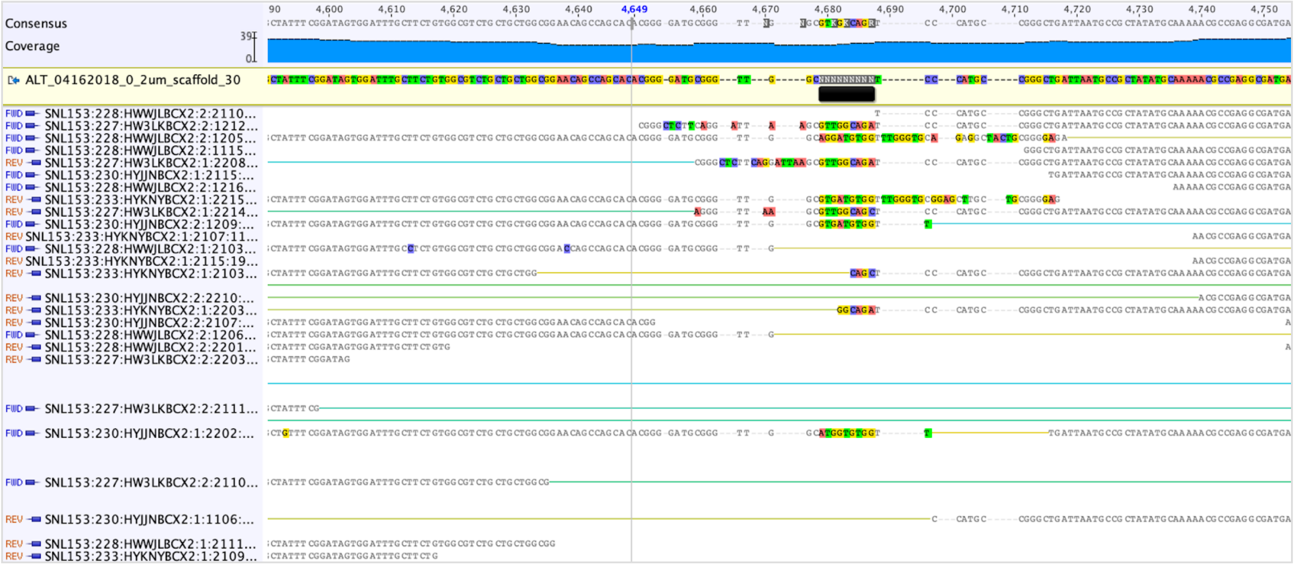

This is what looks like after adding the "-" to the left of the region of confusion.

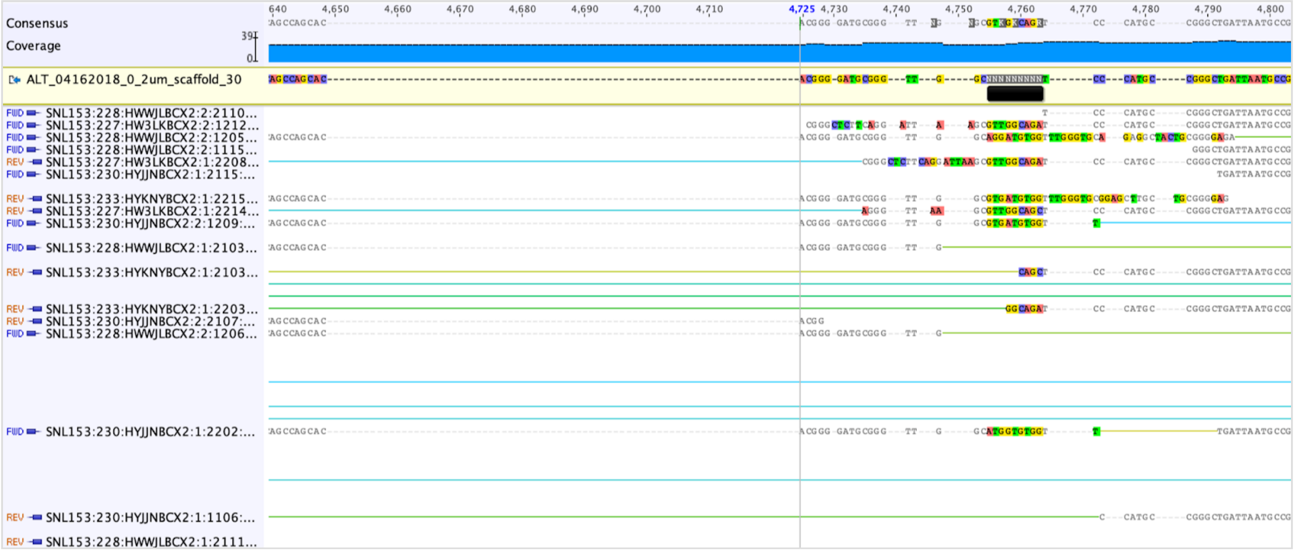


Then we should move reads in this region to the appropriate side:

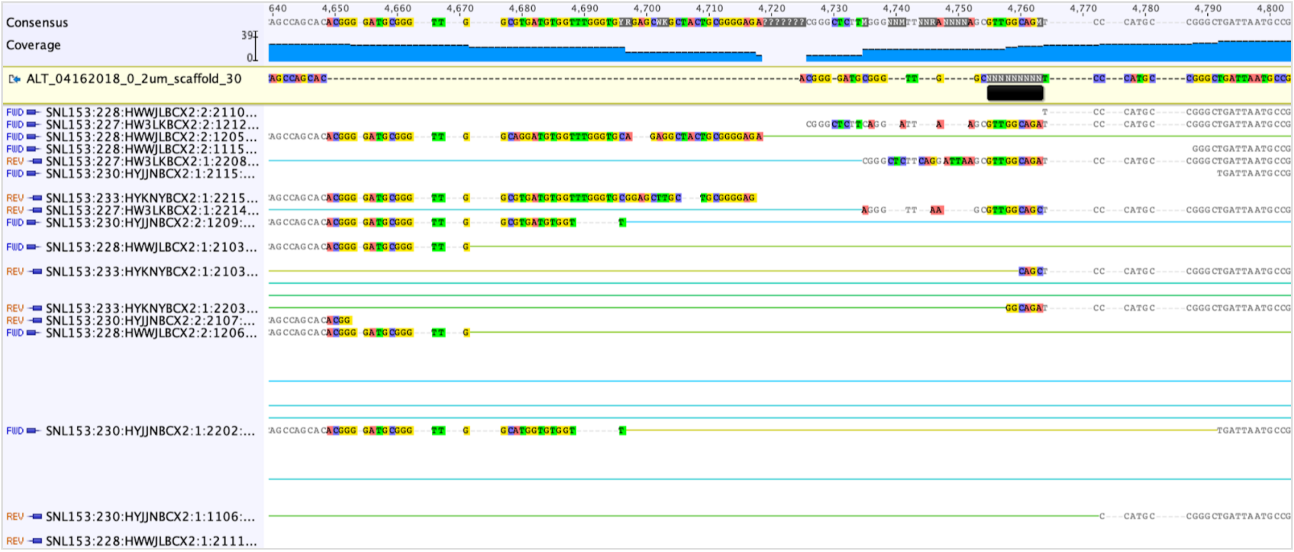


Then delete the columns without any base.

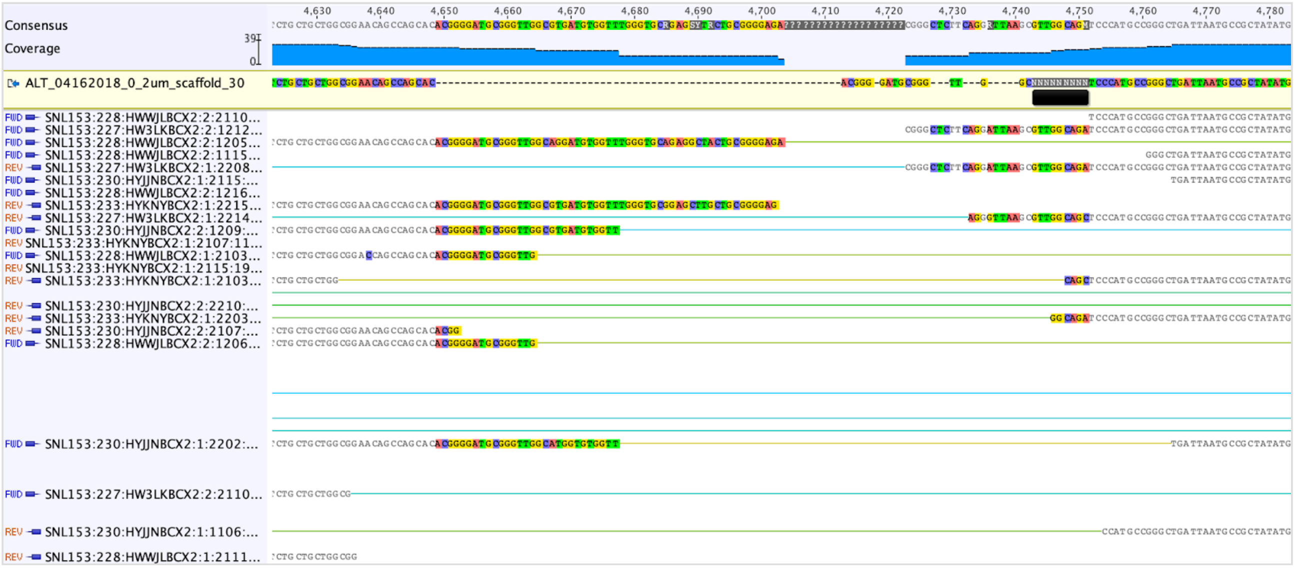


As we can see from the screenshot above, there are sequences shared by the reads that can be copied and pasted into the scaffold sequence to partially fill the gap.

Although the reads in the gap regions shared high sequence similarity, we can also see some single nucleotide variants (SNVs). We choose the consensus sequences shared by majority reads.

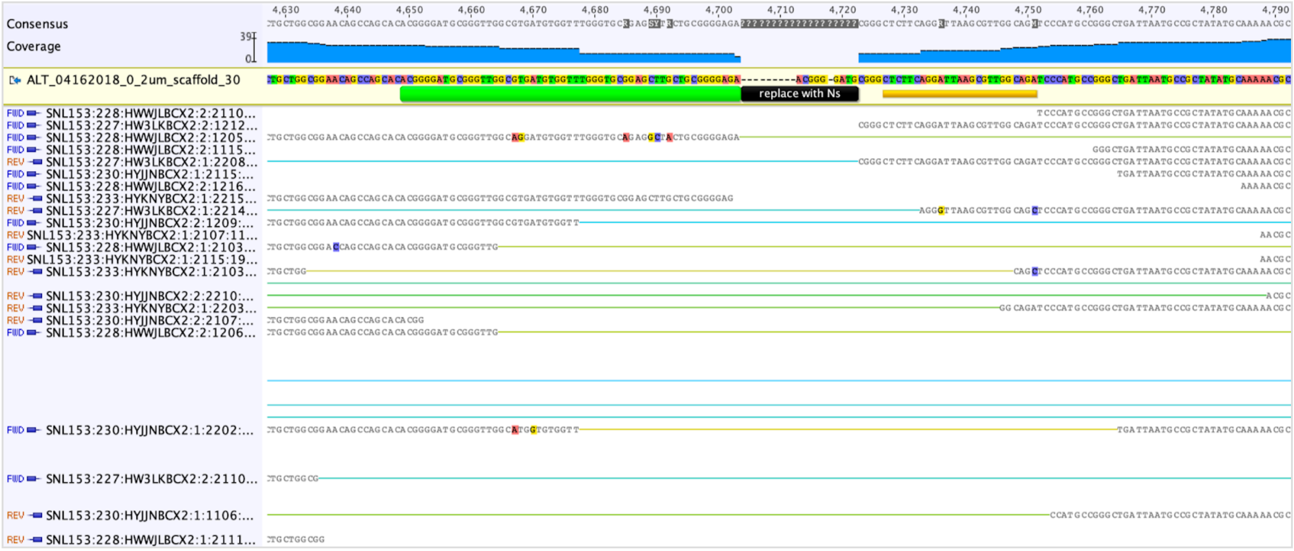


Unsupported consensus sequence should be replaced by Ns.

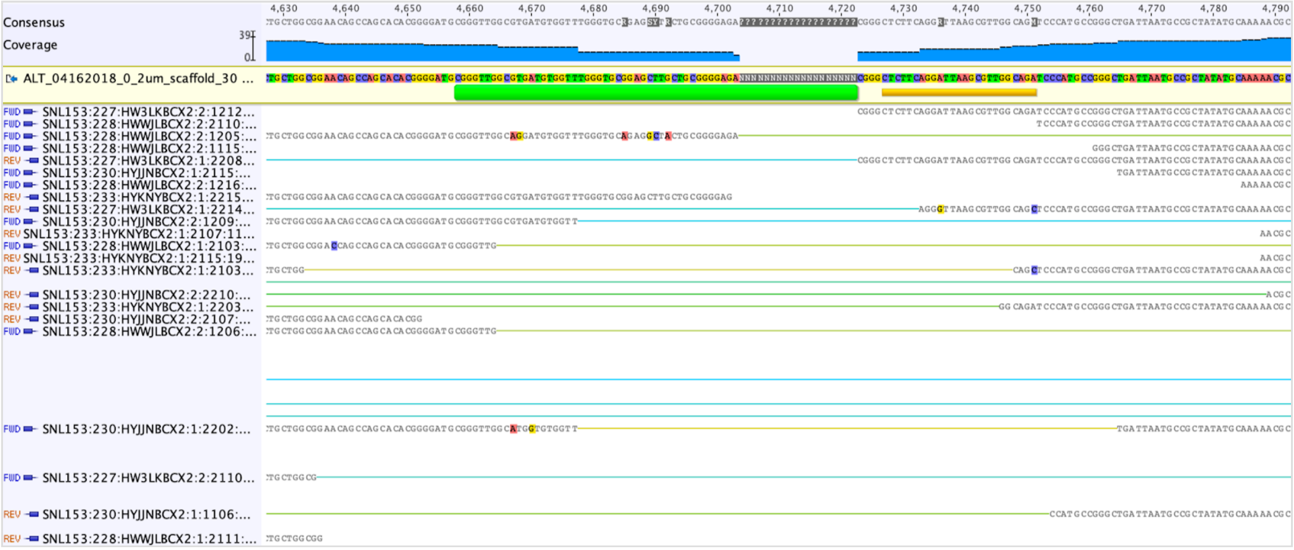


We then save the modified scaffold as "ALT_04162018_0_2um_scaffold_30_modified". We map the unplaced paired reads (same as used to extend scaffold ends, see above) with the "Medium Sensitivity / Fast" to the modified scaffold.

| Name | Sequence Length | # Sequences |
|---|---|---|
| ALT_04162018_0_2um_scaffold_30 – Unmapped sequences from ALT_04162018_0_2um_scaffold_30 | – | 1,043 |
| ALT_04162018_0_2um_scaffold_30 – ALT_04162018_0_2um_scaffold_30 | 369,272 | 41,504 |
| ALT_04162018_0_2um_scaffold_30 | 368,569 | – |
| ALT_04162018_0_2um_scaffold_30_modified | 368,645 | – |

The output mapping profile of the region with the scaffolding gap is shown below:.

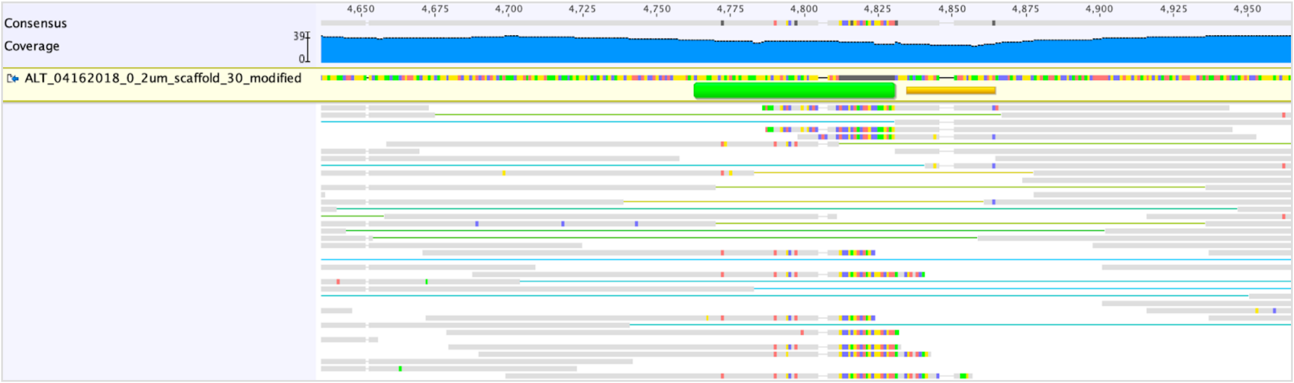


Open the gap and sort the reads as performed above.

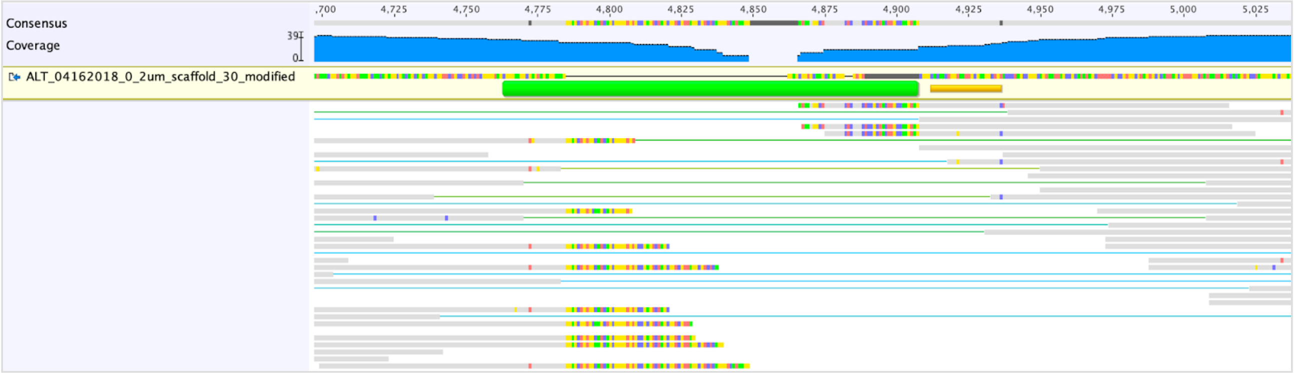

Add consensus sequences shared by the reads to the scaffold. Do not worry about the SNVs we can see now, we will figure out what is happening once the gap is closed.

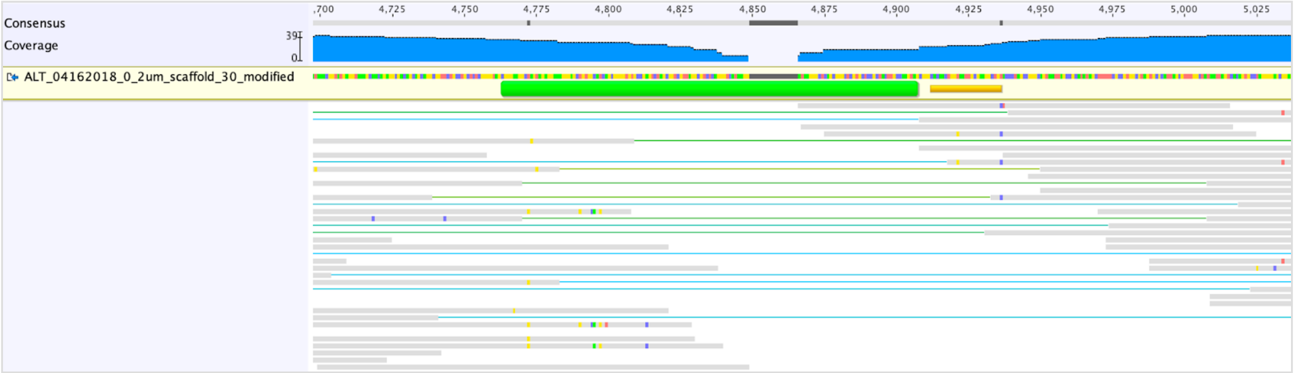


Run of mapping with unplaced paired reads until the gap can be closed:

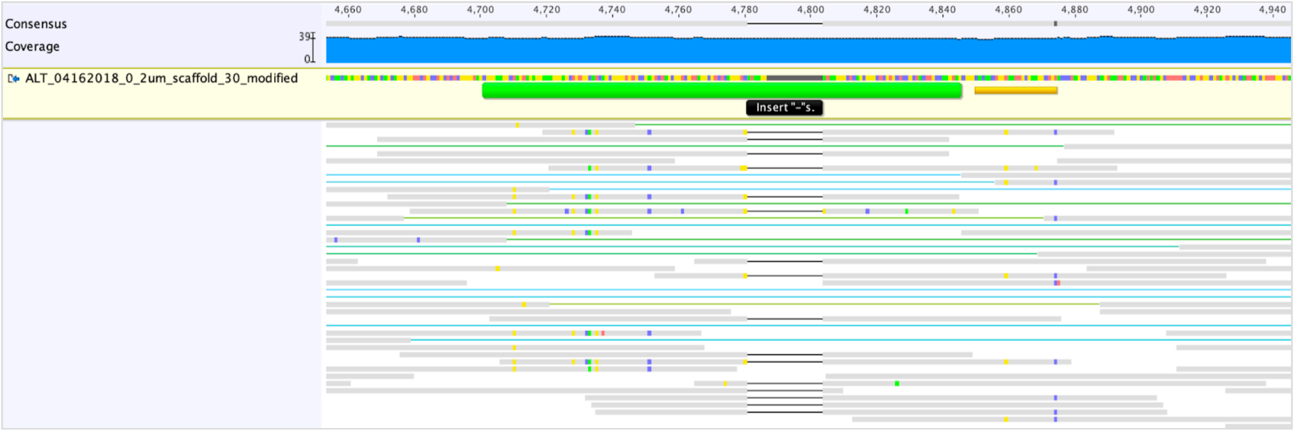


The mapping profiles indicate the gap will be closed once we delete the bases in the scaffolds without read support:

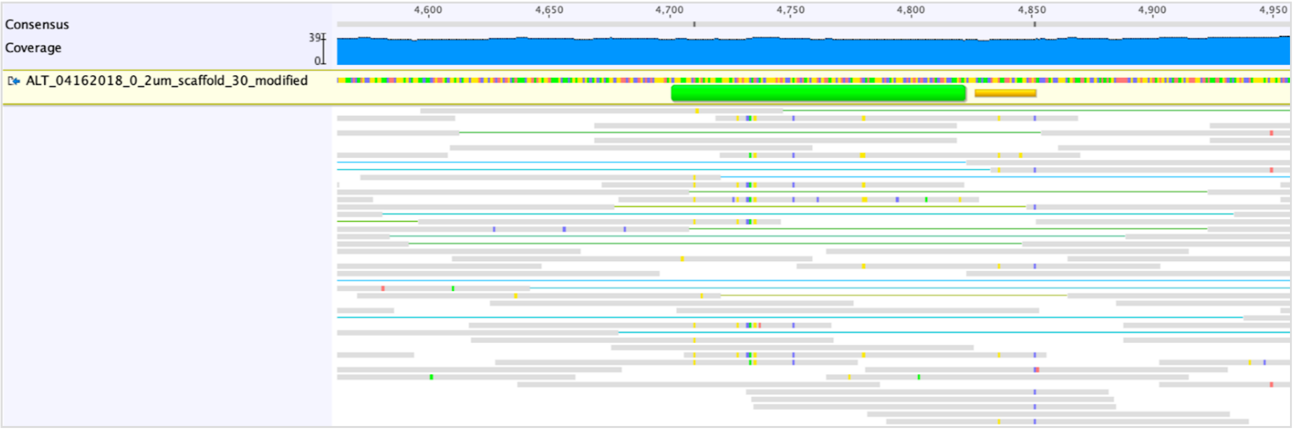


Next, map the reads to the scaffold with the gap closed allowing no mismatch (custom sensitivity, zero mismatch allowed), to see if the region is covered throughout.

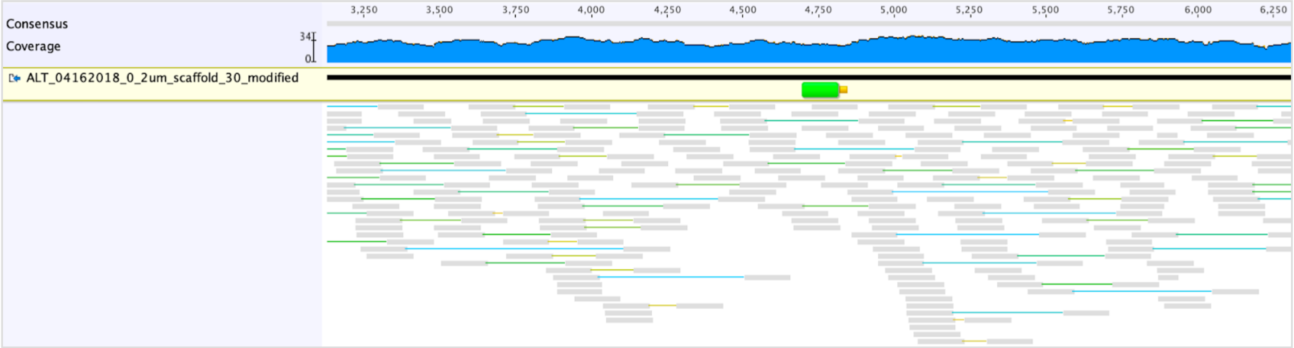


By closing the gap, we obtain the whole protein sequence of the relevant gene, which is confirmed by the BLASTp search.

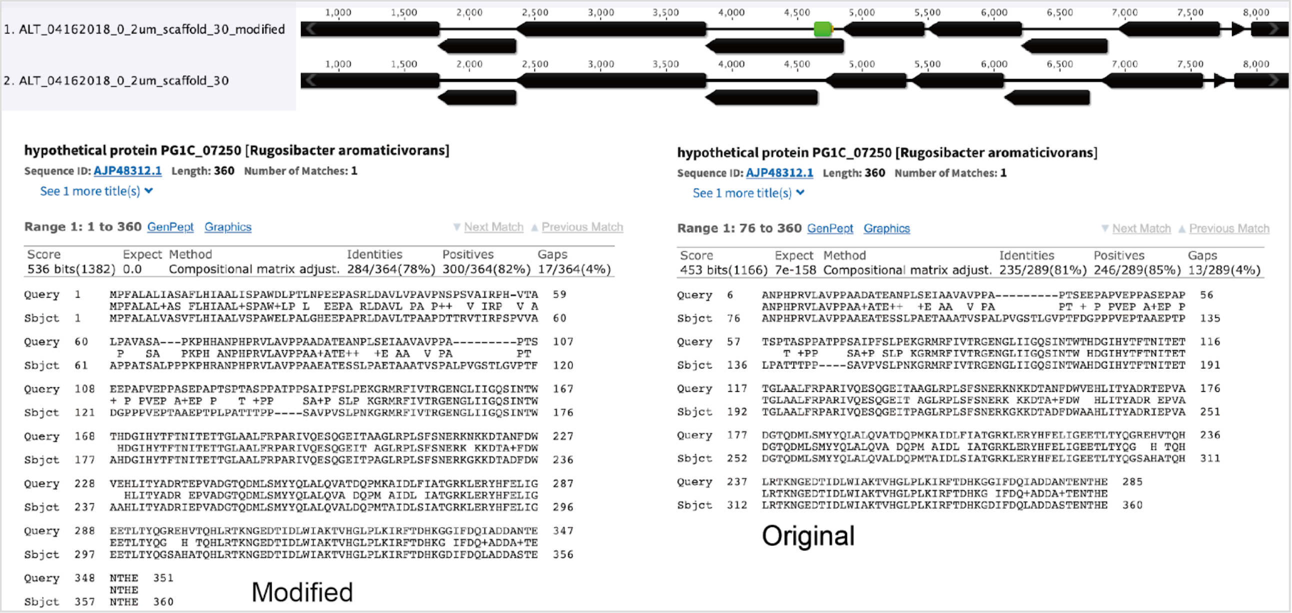


## Conclusion

In this blog, we show how the scaffold extension and scaffolding gap closing can be in Geneious (as an example). Automatic tools should be developed in the future to perform these curation steps.
